# Using cash transfers to promote child health equity: an analysis of Lesotho’s Child Grants Program

**DOI:** 10.1093/heapol/czad044

**Published:** 2023-07-04

**Authors:** Elodie Besnier, Henning Finseraas, Celine Sieu, Kimanzi Muthengi

**Affiliations:** Centre for Global Health Inequalities Research, NTNU Department of Sociology and Political Science, SU Faculty, Norwegian University of Science and Technology, PO box 8900, Torgarden, Trondheim 7491, Norway; NTNU Department of Public Health and Nursing, Faculty of Medicine and Health Sciences, Norwegian University of Science and Technology, PO box 8900, Torgarden, Trondheim 7491, Norway; Centre for Global Health Inequalities Research, NTNU Department of Sociology and Political Science, SU Faculty, Norwegian University of Science and Technology, PO box 8900, Torgarden, Trondheim 7491, Norway; UNICEF Lesotho Country office, 13 UN Road, UN House, Maseru, Lesotho; UNICEF Lesotho Country office, 13 UN Road, UN House, Maseru, Lesotho

**Keywords:** Child health, cash transfer, Lesotho, health equity, social protection, gender equality

## Abstract

Cash transfers (CTs) are increasingly popular tools for promoting social inclusion and equity in children in sub-Saharan Africa. However, less is known about their implications for reducing the health gap between the beneficiary and non-beneficiary children in the community. Using Lesotho’s Child Grants Program (CGP) as a case study, we aim to understand better the potential for CT programmes to reduce the gap in child health in the targeted communities. Using a triple difference model, we examine to what extent CGP improved child health outcomes in eligible households compared with non-eligible households in treatment communities vs control communities and to what extent this effect varied in different population subgroups. We find that the child health gap by beneficiary children’s health outcomes catching-up on the health of non-beneficiary children narrowed but that eduction was not statistically significant. However, such a ‘catch-up’ effect among beneficiaries was observed for selected nutrition outcomes amongst female-headed households and subjective child health assessment for comparatively more food-secure households. This study highlights the potential and limitations of CT programmes like the CGP to address health inequalities in preschool children for selected population subgroups in the community.

Key messages

- While the changes observed over time may suggest a catch-up effect amongst beneficiary households, these effects are not statistically significant.
- The Child Grants Program is associated with a reduction in the gap for selected child health outcomes amongst specific subgroups.
- Cash transfers’ effects on health disparities in children are complex and do not necessarily lead to an overall catch-up effect for beneficiary children.

## Background

Cash transfers (CTs), or non-contributory monetary transfers to individuals or households, have become increasingly popular social protection tools in low- and middle-income countries and particularly in sub-Saharan Africa (UNICEF-ESARO and Transfer Project, 2015; Bastagli *et al.*, 2016). Initially designed to respond to food crises and the human immunodeficiency virus (HIV)/acquired immunodeficiency syndrome epidemic, these programmes are seen as important tools for social inclusion and equity promotion for children in the region (UNICEF-ESARO, Transfer Project, 2015). CT programmes have shown some promising impacts amongst beneficiary children, including effects on health indicators (e.g. health service use and nutrition) and determinants of health (e.g. school attendance, asset ownership and social capital) (Lagarde *et al.*, 2009; Owusu-Addo and Cross, 2014; Bastagli *et al.*, 2016; Bonilla *et al.*, 2017; Pega *et al.*, 2017; Walque, 2017). However, evidence of CT’s impact on health inequalities in children, including the health gap, across the targeted community is lacking (Owusu-Addo *et al.*, 2018; Besnier *et al.*, 2021).

The Empowerment for Health Equity—Lesotho (E4HE Lesotho) project is a mixed-method case study aiming to inform the study of health inequalities and empowerment issues in CTs like Lesotho’s Child Grants Program (CGP). This paper presents the quantitative component of this case study on the CGP and offers to explore the impact of the CGP on the health gap in children under 6 years old.

### Lesotho’s CGP

Lesotho is a landlocked country located in South Africa. When the CGP was established in 2009, Basotho children faced high rates of poverty, food insecurity and the effects of the HIV epidemic, which had fuelled high levels of orphanhood and rising child mortality. This burden was also unevenly distributed, leading to health inequalities in child health (Table 1) (Ministry of Health and Social Welfare—MOHSW/Lesotho and ICF Macro, 2010; UNICEF Lesotho, 2011; UNFPA, 2012).

**Table 1.** Child health inequalities in Lesotho across selected indicators

	Residence		Wealth quintiles		Mother’s education	
	Urban	Rural	Highest	Lowest	Secondary	Did not finish primary school
Child mortality rate (per 1000 live births)	89	110	80	107	88	124
Vaccination coverage in children 12–23 months (in %)	71	59	72	52	66	54
Prevalence of acute respiratory infection in children under age 5 years (in %)	2.7	6.3	1.8	7.9	4.3	7.2
Prevalence of diarrhoea in children under age 5 years (in %)	9.8	11.6	8.6	13.8	9.7	12.9

*Source*: Ministry of Health and Social Welfare—MOHSW/Lesotho, ICF Macro (2010). Lesotho Demographic and Health Survey 2009. MOHSW and ICF Macro., Maseru.

**Figure 1.**
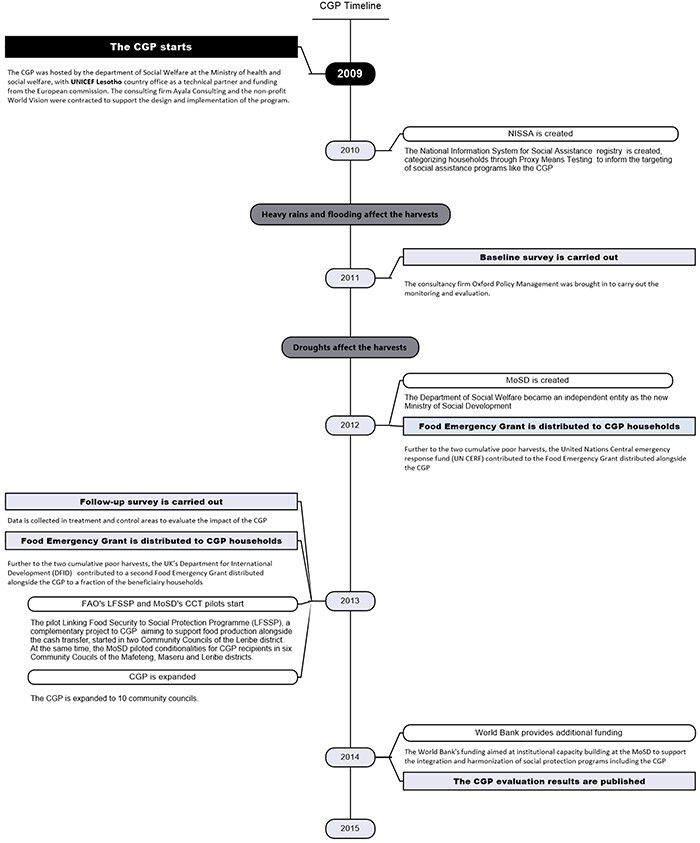
An overview of the CGP and its evaluation

Lesotho’s CGP is an unconditional CT targeting poor and vulnerable households with children under 18 years old. It was hosted by the Department of Social Welfare at the Ministry of Health and Social Welfare (now established as a separate Ministry of Social Development), with UNICEF Lesotho country office as a technical partner and funding from the European Commission (Pellerano *et al.*, 2016). The CGP aims to improve the living standards of orphans and vulnerable children to reduce malnutrition, improve health status and increase school enrolment (Pellerano *et al.*, 2014). Initiated to respond to the impact of the HIV epidemic, the CGP’s scope was broadened to different vulnerabilities affecting children in rural Lesotho (Besnier *et al.*, 2024). In 2009, the CGP covered ∼1250 households in three Community Councils in the districts of Qacha’s Nek, Mafeteng and Maseru. When the follow-up evaluation survey was carried out in 2013, the CGP had been expanded to 2300 households in 10 Community Councils in the five districts of Qacha’s Nek, Maseru, Leribe, Berea and Mafeteng. Figure 1 provides an overview of the programme’s timeline. Targeted communities were primarily located in the rural lowlands and foothills, with limited access to health services and markets (Table 2).

**Table 2.** Health and healthcare usage in areas covered by the CGP

	Treatment areas		Control areas	
	(both beneficiary and non-beneficiary households)		(both eligible and non-eligible households)	
	2011	2013	2011	2013
Average travelling time to the nearest health clinic (in hours)	2.8	2.7	2.9	2.7
Proportion of children under 3 years old fully immunized	[Data not collected]	49.2	[Data not collected]	57
Proportion of children (0–5 years old) who suffered any illness in the last months	38.9	31.4	36.7	45.3
Proportion of children (0–5 years old) for whom any money was spent for healthcare in the last 3 months^a^	12.7	13.6	17.9	17.9
Average amount spent on child healthcare^a^ children (0–5 years old) in the last 3 months (in Maloti)	35	42.6	59	44.3

a Primary healthcare in public facilities is free in Lesotho. The expenditure presented here would include transport, over-the-counter drugs and additional services not part of the basic primary healthcare package and, in fewer cases, fees in private facilities.

Adapted from: Pellerano L, Moratti M, Jakobsen M, Bajgar M, Barca V. 2014. The Lesotho Child Grants Programme Impact Evaluation: Follow-up Report. UNICEF Lesotho (with EU funding and technical support from FAO), Maseru.

CGP households were selected through a mix of Proxy Means Testing (based on a community census)^1^ and a validation process by representatives of the community. Only households with children identified as ultra-poor and very poor are eligible for the CGP. Beneficiary households received 360 maloti quarterly (about USD 30)—an amount later adapted from 300 to 750 maloti according to the number of children in the household (Pellerano *et al.*, 2014; 2016). Although the CGP did not apply strict conditionality to beneficiary households, the implementation of the programme included a strong messaging (or soft conditionality) that the transfer was to be used for the children (Pellerano *et al.*, 2014).

The programme’s theory of change^2^ articulated that by reducing poverty and addressing the underlying causes of poverty of vulnerable households, the CGP would reduce inequalities while enabling households to make different time and investment decisions, participate in economic activities and enhance future productivity. The 2014 evaluation found promising effects of the CGP amongst beneficiaries regarding economic indicators, child health outcomes and determinants of health (Pellerano *et al.*, 2014). In addition, the local economy-wide impact evaluation found significant economic spillover across the communities where the CGP was implemented (Thome *et al.*, 2016). Finally, Carraro and Ferrone (2019) found that food security in non-beneficiary households close to beneficiaries had improved through programme spillover. However, the programme’s effects on child health inequalities between different groups in these communities and its implications for vulnerable groups have not been studied.

### CTs, child health and gender

CTs are the fastest-growing type of safety net programme on the African continent (Beegle *et al.*, 2018). CTs have been found to improve child health and development outcomes amongst beneficiaries. However, evidence syntheses and reviews have found mixed and occasionally contradicting results as to CTs’ impact on individual outcomes, suggesting that they vary from one CT to the other (Bastagli *et al.*, 2016; Walque, 2017; Beegle *et al.*, 2018). Some CT programmes have also shown that programme impact on child health may vary by gender (of the child or the head of the household) (Yoong *et al.*, 2012; Bastagli *et al.*, 2016), but the evidence on differentiated child health impact between vulnerable groups remains limited (Bastagli *et al.*, 2016). Besides their direct impact on child health, CTs have also been associated with improving determinants of child health amongst beneficiaries, such as a reduction in monetary poverty or improved food security (UNICEF-ESARO, Transfer Project, 2015; Bastagli *et al.*, 2016; Owusu-Addo *et al.*, 2018).

Given women’s role in childcare, many CTs have targeted women specifically. Indeed, selected markers of women’s empowerment and status (e.g. women’s decision-making power and control over economic resources and assets) are associated with improved child health outcomes (Duflo, 2012; Richards *et al.*, 2013; Kuruvilla *et al.*, 2014; Carlson *et al.*, 2015; Cunningham *et al.*, 2015; Taukobong *et al.*, 2016; Thorpe *et al.*, 2016). Women might be more likely to invest in family-friendly goods beneficial to children (Yoong *et al.*, 2012; Richards *et al.*, 2013). Yet, the impact of CTs targeting women on child health is disputed. Few studies compare the effects of targeting women vs men, and findings on the respective child health impact were mixed (Yoong *et al.*, 2012; Bastagli *et al.*, 2016). Besides their instrumental role in child development, women and girls have been targeted directly to empower female beneficiaries (Yoong *et al.*, 2012; Bastagli *et al.*, 2016). In this field, selected CTs have seen improved women’s social and economic empowerment (such as greater autonomy and influence over households’ decisions). However, the evidence is, again, mixed (Bastagli *et al.*, 2016).

As these findings show, CTs can reduce vulnerabilities in beneficiary children and their caregivers, which makes these programmes promising for promoting health equity. In addition, evidence of the indirect effects of CTs on various socioeconomic determinants of health beyond the beneficiary group would further support this hypothesis (Angelucci and De Giorgi, 2009; Angelucci *et al.*, 2010; Thome *et al.*, 2016; Carraro and Ferrone, 2019). However, their actual effects on health (e.g. gap or gradient) remain understudied (Owusu-Addo *et al.*, 2018; Besnier *et al.*, 2021).

Our study of the role of health equity and economic empowerment in Lesotho’s CGP shows that both were integrated to some degree in the CGP from its early phases (Besnier *et al.*, 2024). Second, as the economic empowerment of vulnerable groups was seen as a strategic objective and mechanism of action of the CGP, we would expect to see specific vulnerable groups (e.g. female-headed households and more deprived households) benefitting more than comparatively less vulnerable groups (Besnier *et al.*, 2024).

### Objective

Using the early phases of the CGP as a case study (i.e. 2009–2013), this study aims to better understand the potential for such programmes to reduce inequalities in child health, especially the health gap, in the targeted communities. More specifically, this study answers the following research questions:

- What were the effects of the CGP on the gap in health outcomes between the beneficiary and non-beneficiary households with children under 6 years old?
- Did comparatively more vulnerable groups at baseline see a reduction in this gap, thanks to the CGP?

### Scope

To better capture the effects of the CT alone, this study focuses on the early phases of the programme before complementary interventions (Cash Plus) were piloted. The data used for the analysis were collected in 2011 and 2013. The study takes into account that the cash ‘Emergency Food Grant’ (Figure 1) was distributed to eligible households in control and treatment areas in 2012–2013 (although the amount distributed varied from one household to the next), as it overlaps with the CGP.

Due to the short timespan covered, the broader socioeconomic and political context (i.e. structural determinants of health) is considered constant in the period of interest.

### Conceptual background

In their more neutral definitions, health inequalities refer to observable differences in health between individuals or groups (Kawachi *et al.*, 2002). However, this term is often used interchangeably with the term health (in)equity, defined as ‘avoidable’ health inequalities (Graham, 2004; Whitehead and Dahlgren, 2006; Commission on Social Determinants of Health, 2008). The various definitions of health (in)equity rely on two key ideas. First, one’s health is not defined by biology or choices alone but depends on their wider environment (known as determinants of health). Second, some of the health inequalities resulting from disparities in the distribution of these determinants are socially produced and, therefore, unfair (Dahlgren and Whitehead, 1991; Kawachi *et al.*, 2002). In this study, we use the general term ‘health inequalities’ when referring to all disparities in health (regardless of their cause) and ‘health inequities’ to highlight the un(fairness) and socially constructed nature of health inequalities.

By addressing disparities in these determinants, CT programmes like the CGP could contribute to reducing these inequities. Based on the CGP’s theory of change and building on the World Health Organization Commission on the Social Determinants Health (CSDH)’s conceptual framework (Solar O and Irwin A, 2010; Pellerano *et al.*, 2012), this unconditional CT programme could affect the health gap in child health outcomes between beneficiary and non-beneficiary both directly and indirectly. The conceptual background for the whole E4HE Lesotho case study is available in the E4HE qualitative studies (Besnier *et al.*, 2024). This conceptual background was used to support data analysis in the qualitative part of this case study, whose results informed the quantitative analysis presented here (see the section on Selection of variables).

### Direct effects on the health gap

The CGP provides supplementary income to ultra-poor and very poor households, thus reducing poverty and the economic inequities in the community (Pellerano *et al.*, 2012). Hence, it affects beneficiary households’ socioeconomic position as well as their children’s material circumstances (e.g. clothing and access to food). Via these pathways, it directly contributes to child health improvements and, through the reduction in social and economic disparities with non-beneficiaries, may reduce the gap in health outcomes between children in the beneficiary and non-beneficiary households. However, since non-beneficiary households are better-off at the start of the programme, the reduction in the health gap will only occur as long as other factors do not make non-beneficiary children’s health improve faster. Suppose the theory that female recipients may invest the transfer in more family-friendly goods than male recipients is confirmed, the programme’s effect on children’s outcomes should be larger in female-headed beneficiary households. If the CGP effectively addresses multiple vulnerabilities and empowers vulnerable groups, the most vulnerable groups should see a greater impact.

The CGP may also affect child health inequalities in the community through another pathway. As beneficiary households spend the CGP funds and can re-enter sharing networks in their community, non-recipient households may also see their incomes increase. Hence, the CGP may further affect the structure of health inequities in the treatment communities by modifying the socioeconomic position and material circumstances of non-beneficiary children in the community. This is a direct effect since it follows directly from the CT. Depending on the size of this effect on non-beneficiary households compared with the effect on beneficiary households, this effect may increase the health gap in the community.

### Indirect effects on the health gap

The CGP may also affect child health and health inequities indirectly through economic empowerment. As the CGP’s theory of change illustrates, by providing additional financial resources, CTs can improve beneficiary households’ access and control over such resources while reducing the effect of shocks or other constraints. As a result, households can invest in social and human capital to improve their agency (Pellerano *et al.*, 2012). Additionally, following the CSDH framework’s description of the mechanisms through which income affects health (Solar and Irwin, 2010), the CGP could also affect the agency of beneficiary households (and particularly their female members), if not that of the community as a whole. By reducing stress caused by poverty and increasing their ability to cope with risks and shocks, CTs may affect the psychosocial determinants of health in the beneficiary households (Bastagli *et al.*, 2016; Molyneux *et al.*, 2016; Samuels and Stavropoulou, 2016; Zimmerman *et al.*, 2021). Previous literature on CTs also shows that some programmes can affect power relations and bargaining powers within the household, which can improve both the control over resources and the agency of children’s caregivers (especially women) (Bastagli *et al.*, 2016; Bonilla *et al.*, 2017). Second, the programme’s evaluation highlights how the CGP had modified beneficiary households’ participation and place in the community’s support and sharing networks, thus potentially affecting social cohesion (Pellerano *et al.*, 2014). By affecting these intermediary determinants of health in disadvantaged households, the CGP may affect the distribution of selected child health outcomes in the community, thus affecting the health gap between the beneficiary and non-beneficiary households and/or modifying the structure or gradient of such outcomes.

### Hypothesis

This study will test the ‘catch-up’ hypothesis as to how the CGP might have affected the health gap between beneficiary and non-beneficiary children in targeted communities. This hypothesis is articulated as follows:

- Due to the CGP, the gap in health outcomes between children in eligible and non-eligible households in treatment communities is reduced compared with the gap in control communities. The beneficiary group’s health improves at a faster path than non-beneficiaries’, allowing children receiving the CGP to ‘catch up’ on their health disadvantage. This hypothesis implies that the programme mainly impacts the health of the targeted group, either directly or indirectly.
- If we assume that CGP reduces multiple vulnerabilities and empowers vulnerable groups as intended, then this catch-up effect should be larger in households that were more vulnerable at baseline (e.g. female-headed households and poorer households).

## Methodology

### Data and study design

To better evaluate the impact of the CGP, the programme was initially designed as a randomized controlled trial. Within each of the 10 Community Councils covered, electoral divisions were randomly assigned to the treatment group (where eligible households received the CGP) or control group (where households were divided between eligible and non-eligible, but the CGP’s implementation was delayed until the end of the evaluation). The evaluation of the CGP included household survey data collected at baseline and follow-up from both eligible and non-eligible households in treatment and control areas. The evaluation was led by Oxford Policy Management, while Sechaba Consultant collected the survey data. They surveyed 3054 households in 2011 (at baseline) and 2300 households at the same time of the year in 2013 (follow-up).^3^ The household questionnaire covered 22 themes, including households’ general characteristics, economic characteristics and activities, consumption, food security, community networks, individual member’s demographics, health, child education, adult labour participation and child labour and time use (Pellerano *et al.*, 2014). Survey instruments are available on the Transfer Project website (Oxford Policy Management, 2011; 2013). The overall sample attrition was low (6%), and the sample was generally balanced between control and treatment areas (Pellerano *et al.*, 2014). As this study focuses on a subset of this sample (households with children under 6 years old), our sample includes 1532 households (Table 3).

**Table 3.** Distribution of households by eligibility and areas

	Control areas Frequency (%)	Treatment areas Frequency (%)	Total
Non-eligible	210 (31.72)	248 (28.51)	458
Eligible	452 (68.28)	622 (71.49)	1074
Total	662	870	1532

### Data sources

This study relies on the ‘Lesotho-Child Grant Programme data’, available from the Transfer Project data portal (hosted by the University of North Carolina at Chapel Hill—Carolina Population Center—UNC Carolina Population Center, 2021).

### Selection of variables

The selection of variables for the model was guided by the outcomes of our previous E4HE qualitative study analysing how CGP stakeholders had defined and operationalized the concept of health equity (Besnier *et al.*, 2024). In this study, health was primarily defined as access to healthcare and, to a lesser extent, as nutrition and health status, as reported by the caregiver. For this model, we examine our hypothesis using four child health outcomes: (1) whether any of the children have been ill over the last 30 days, (2) whether the household has spent money on healthcare (including transport or medicines) over the last 30 days, (3) self-assessment by the adult respondent of the children’s health status in the household (categorized here as ‘good’ or not) and (4) children’s food security (as a proxy for nutrition status), measured by three variables, such as a child not going to bed hungry, eating fewer meals or eating smaller meals in the last 3 months. All variables were coded as 1 for good health and nutrition and 0 for other answer categories. Due to a lack of data at baseline, anthropometric variables (weight between birth and 2 years old) and immunization records were excluded. All outcomes refer to children below the age of 6 years old. Eligibility to CGP is used as a proxy for socioeconomic status, as the programme targeted the poorest households.

To explore the effect of the CGP on vulnerable subgroups, we based our selection of vulnerability variables on how CGP stakeholders had defined vulnerabilities but also economic empowerment and gender issues, in the E4HE qualitative studies (Besnier *et al.*, 2024). By discussing the role of economic empowerment, stakeholders identified households’ characteristics that should be particularly affected by the CGP. The first variable we identified for the subgroup analysis is food security at baseline (as a proxy for access to economic resources). Stakeholders had also explained the *de facto* role of gender (understood primarily as mothers and female heads of households) in the programme due to the increased vulnerabilities of female-headed households and women’s role in childcare. To explore the CGP’s potential impact on gendered vulnerabilities, we include the gender of the head of the household (whether the head of the household is a woman or a man) as our second vulnerability variable.

### Analysis

First, we provide an overview of households’ background characteristics by areas (treatment vs control), eligibility and gender of the head of household. Then, we estimate the impact of the CGP on the health gap using a triple differences (DDD) model. As the following equation shows, we compare the changes in child health outcomes between baseline and follow-up for beneficiaries and non-beneficiaries in treatment areas with the changes observed between eligible and non-eligible in control areas. Our estimates are intention-to-treat effects.


$$\begin{aligned}HEALT{H_{it}} =\,& {\beta _1}\,{A_i} + \,{\beta _2}\,ELI{G_i} + {\beta _3}\,{T_t}\, + {\beta _4}\,{T_t}*\,ELI{G_i}\, \\&+ \,{\beta _5}\,{A_i}*{T_t}\, + {\beta _6}\,{A_i}*\,ELI{G_i}\, \\&+ {\beta _7}\,{A_i}*{T_t}*\,ELI{G_i}\, + {\varepsilon _{it}}\end{aligned}$$

where *i* indexes household and *t* indexes baseline (*t* = 0) or endline (*t* = 1). *HEALTH* is our measure of child health. *A* represents the area’s participation and takes the value of 1 for treatment communities and 0 for control communities. *ELIG* is a binary indicator of eligibility to receive the CGP. Survey rounds are indicated with *T*, and it takes the value of 1 for endline and 0 for baseline, while *ε* is the error term. All independent variables are binary indicators. As the randomized design of the programme controls for unobserved variables, we do not include control variables. Since our child health and nutrition outcomes tend to be highly correlated, each ordinary least square regression was run separately.

The catch-up hypothesis says that the health gap between children in beneficiary and non-beneficiary households (i.e. eligible and non-eligible households in the treatment communities) should decrease. The DDD estimate tells us whether the difference between eligible and non-eligible, from baseline to follow-up, is different in the treatment areas compared with the control areas. This estimate is the causal effect of programme eligibility, assuming that the difference between eligible and non-eligible from baseline to follow-up in the control area is a valid counterfactual development for the same difference in the treatment area.

To explore the effect of the CGP on the health gap between different subgroups groups, we subset the analysis by the child food security status of the household at baseline (as a proxy for access to economic resources) and by the gender of the head of households. For the analysis by food security status, the nutrition outcomes were not included as they overlap with the variable used to assess access to economic resources.

## Results

### Background characteristics

Table 4 provides an overview of households’ background characteristics at baseline in control and treatment areas. To confirm that eligible households were more disadvantaged than non-eligible households, we estimated the baseline balance between eligible and non-eligible households (Table A1a in Appendix 1–4). This confirms that eligible households tended to be worse off on child health and nutrition indicators than non-eligible households (as expected).

**Table 4.** Descriptive statistics in treatment and control areas

	Control areas				Treatment areas				
	(1)				(2)				
	Non-eligible		Eligible		Non-eligible		Eligible		*t*-test difference
	*N*	Mean/SE	*N*	Mean/SE	*N*	Mean/SE	*N*	Mean/SE	(1)-(2)
Male-headed households	92	0.641	215	0.530	110	0.800	295	0.559	−0.061*
		[0.050]		[0.034]		[0.038]		[0.029]	
Adult education achievement	105	0.351	226	0.303	124	0.290	311	0.292	0.027*
		[0.022]		[0.015]		[0.021]		[0.012]	
Households own or cultivate land in the past year	105	0.905	226	0.823	124	0.903	311	0.907	−0.057**
		[0.029]		[0.025]		[0.027]		[0.017]	
Households have access to a piped water supply source	105	0.505	226	0.571	124	0.565	311	0.498	0.033
		[0.049]		[0.033]		[0.045]		[0.028]	
Children <3 years old have a bukana card^4^	105	0.752	226	0.611	124	0.726	311	0.614	0.010
		[0.042]		[0.033]		[0.040]		[0.028]	
Children in the household have a birth certificate	105	0.229	226	0.115	124	0.153	311	0.164	−0.010
		[0.041]		[0.021]		[0.032]		[0.021]	
Has received a CT/public assistance in the last year	105	0.105	225	0.102	124	0.161	311	0.100	−0.014
		[0.030]		[0.020]		[0.033]		[0.017]	
Adults in the household have a passport^5^	104	0.692 [0.045]	223	0.453 [0.033]	123	0.707 [0.041]	308	0.477 [0.029]	−0.014
No child illness in the last 30 days	105	0.602	226	0.627	124	0.510	311	0.594	−0.032
		[0.045]		[0.030]		[0.043]		[0.026]	
Healthcare spending on children in the last 3 months	105	0.196	222	0.194	124	0.295	307	0.185	0.049
		[0.037]		[0.025]		[0.040]		[0.021]	
Self-assessed child health status	104	0.949	225	0.928	121	0.944	308	0.891	−0.022
		[0.020]		[0.017]		[0.019]		[0.017]	
No child going to bed hungry in the last 3 months	105	0.762	226	0.615	124	0.798	311	0.691	0.029
		[0.042]		[0.032]		[0.036]		[0.026]	
No child eating fewer meals in the last 3 months	105	0.543	226	0.235	124	0.556	311	0.322	−0.060*
		[0.049]		[0.028]		[0.045]		[0.027]	
No child eating smaller meals in the last 3 months	105	0.514	226	0.226	124	0.524	311	0.302	−0.056
		[0.049]		[0.028]		[0.045]		[0.026]	

The values displayed for *t*-tests are the differences in the means across the groups.

** and * indicate significance at the 5 and 10% critical levels.

Ahead of the subgroup analysis, we also estimated the baseline balance between food-insecure and food-secure households (Table A1b in Appendix 1) and between female-headed and male-headed households (Table A1c in Appendix 1). Food-secure households at baseline tend to score better on a number of socioeconomic and health characteristics (e.g. adult education, children having a bukana card and a birth certificate and child illnesses) although these differences were not always statistically significant—possibly because of the small sample of food-secure households. Looking at the gender of the head of the households, male-headed households also tend to score better than female-headed households on socioeconomic and health characteristics.

### CGP’s effect on child health disparities

Table 5 presents the results of the DDD model for our sample. The final coefficient (Area_Treat*Eligible*F-up—the DDD estimate) tells us whether the change from baseline to follow-up is different for the eligible vs the non-eligible households in treatment areas compared with control areas. If the CGP led to catch-up in child health, we should expect these coefficients to be positive and significant. The coefficients are positive, suggesting that the CGP improved child health and nutrition outcomes for beneficiaries compared with non-beneficiaries (as compared with trends in the control areas). We find that the CT improved child health outcomes from 5 to 13 percentage points (columns 1–3) and nutrition outcomes from 3 to 5 percentage points. However, none of them are statistically significant, meaning that we cannot reject the null hypothesis for any of these estimates. The Area_Treat*F-up coefficient (equivalent to a difference-in-difference estimate comparing changes in non-eligible households in treatment and control communities) confirms that spillover effects did not interfere with the effects observed here (see also Appendix 2, Tables A2a–c). The follow-up coefficients show that there was a worsening of these outcomes between baseline and follow-up but that such effects varied across treatment and control areas, which may have affected the impact of the CGP and our ability to derive robust conclusions on the effect of the programme.

**Table 5.** DDD model results for child-level outcomes in household-level analysis (outcomes are coded so that a positive score means good nutrition and health in children under 6 years old)

	No child illness in the last 30 days	Healthcare spending on children in the last 3 months	Self-assessed child health status	No child going to bed hungry in the last 3 months	No child eating fewer meals in the last 3 months	No child eating smaller meals in the last 3 months
Treatment areas	−0.092	0.099*	−0.005	0.036	0.014	0.010
	(0.062)	(0.054)	(0.028)	(0.055)	(0.066)	(0.066)
Eligible	0.025	−0.002	−0.021	−0.147***	−0.308***	−0.289***
	(0.054)	(0.044)	(0.026)	(0.053)	(0.056)	(0.056)
Follow-up	−0.044	0.109**	−0.044	−0.019	−0.124**	−0.143**
	(0.063)	(0.052)	(0.034)	(0.051)	(0.057)	(0.056)
Elig*F-up	−0.008	−0.088	0.034	0.062	0.204***	0.201***
	(0.075)	(0.062)	(0.042)	(0.064)	(0.070)	(0.069)
Area_Treat*F-up	0.034	−0.149**	−0.034	0.017	−0.009	0.002
	(0.082)	(0.071)	(0.050)	(0.070)	(0.081)	(0.080)
Area_Treat*Eligible	0.059	−0.108*	−0.032	0.040	0.073	0.067
	(0.074)	(0.063)	(0.037)	(0.069)	(0.077)	(0.077)
Area_Treat*Eligible*F-up	0.047	0.128	0.050	0.042	0.054	0.031
	(0.098)	(0.082)	(0.059)	(0.087)	(0.098)	(0.097)
Constant	0.602***	0.196***	0.949***	0.762***	0.543***	0.514***
	(0.045)	(0.037)	(0.020)	(0.042)	(0.049)	(0.049)
Observations	1532	1524	1524	1531	1532	1532
*R*-squared	0.008	0.011	0.007	0.023	0.042	0.036

Standard errors clustered on households are in parentheses.

***p < 0.01, **p < 0.05, *p < 0.1.

We suspect that the statistical power of the analysis may have been affected by contradictory trends at the local level. Hence, we analysed these results by districts. Indeed, in our qualitative study, some of the stakeholders suggested that vulnerabilities and constraints (e.g. access to services and economic opportunities) may differ from one area to the next. We performed an analysis of variance, adjusting *P*-values for multiple testing using the Bonferroni correction, to confirm whether some of the districts showed statistically significant differences in the child health outcomes of interest. This test confirmed that the populations in the districts of Maseru, Leribe and Berea (as a whole or eligible households in particular) showed such differences. Therefore, to check whether our general estimates were affected by contradictory trends in these specific districts, we tested the model by removing one district at a time as a robustness test. The DDD results (see Tables A3a–f in Appendix 3) showed overall similar estimates as those presented in Table 5. However, the findings for child health spending show that their statistical significance is affected by specific districts (Maseru and Leribe). These variations may be the result of district-specific trends. However, the sample sizes of individual districts (and related lack of statistical power) prevented us from exploring this further.

### Subgroup analyses

To test the impact of the CGP on different vulnerable groups, we ran a subgroup analysis according to the child food security level at baseline (Table 6) and the gender of the head of the household (Table 7). We only report the DDD estimates (full tables are available in Appendix 4, Tables A4a, A4b).

**Table 6.** DDD model results for child-level outcomes in household-level analysis by food security status at baseline (outcomes are coded so that a positive score means good nutrition and health in children under 6 years)

	Households where children ‘had to’ eat fewer meals in the last 3 months at baseline (0)			Households where children ‘did not’ have to eat fewer meals in the last 3 months at baseline (1)		
	Illness in the last 30 days	Healthcare spending in the last 3 months	Self-assessed health status	Illness in the last 30 days	Healthcare spending in the last 3 months	Self-assessed health status
Area_Treat*Eligible*F-up	0.061 (0.130)	0.205* (0.108)	−0.110 (0.083)	−0.035 (0.159)	0.076 (0.133)	0.198** (0.088)
Observations	976	972	970	562	558	560
*R*-squared	0.018	0.014	0.005	0.015	0.016	0.025

Robust standard errors are in parentheses.

**p < 0.05, *p < 0.1.

**Table 7.** DDD model results for child-level outcomes in household-level analysis by gender of the head of the households (outcomes are coded so that a positive score means good nutrition and health in children under 6 years)

	Female-headed households			Male-headed households		
	Illness in the last 30 days	Healthcare spending in the last 3 months	Self-assessed health status	Illness in the last 30 days	Healthcare spending in the last 3 months	Self-assessed health status
Area_Treat*Eligible*F-up	−0.045 (0.170)	−0.101 (0.148)	0.128 (0.107)	0.136 (0.130)	0.210* (0.109)	0.031 (0.078)
Observations	572	570	568	852	846	848
*R*-squared	0.002	0.026	0.010	0.016	0.011	0.008
	**Going to bed** **hungry in the last** **3 months**	**Eating fewer** **meals in the last** **3 months**	**Eating smaller** **meals in the last** **3 months**	**Going to bed** **hungry in the last** **3 months**	**Eating fewer** **meals in the last** **3 months**	**Eating smaller** **meals in the last** **3 months**
Area_Treat*Eligible*F-up	0.031	0.366**	0.304*	0.056	−0.154	−0.168
	(0.162)	(0.177)	(0.175)	(0.116)	(0.130)	(0.127)
Observations	572	572	572	851	852	852
R-squared	0.027	0.066	0.051	0.027	0.029	0.025

Robust standard errors are in parentheses.

**p < 0.05, *p< 0.1.

Table 6 shows positive DDD estimates for two outcomes: healthcare spending on children (benefitting food-insecure households) and self-assessed health (in favour of more food-secure households). However, looking at the differences between the two groups, only the effect on self-assessed health is statistically significant. This implies that unlike comparatively more vulnerable households, more food-secure households saw a catch-up effect on how they assessed the health of their children.

Looking at Table 7, we see positive DDD estimates for health outcomes for male-headed households and for female-headed households regarding nutrition outcomes. Interestingly, the other group also systematically observed an opposite trend, as the negative DDD estimates for child health outcomes amongst female-headed households and those for child nutrition outcomes amongst male-headed households show. These opposite trends may explain why the general results in Table 5 did not show any statistically significant results. As the two groups are almost evenly divided in our sample (40% male-headed households vs 60% female-headed households), their opposite effects may be cancelling each other. The results for child healthcare spending in Table 7 should be interpreted with caution, as the difference between male- vs female-headed households for this estimate is only significant at a fairly high *P* value (*P* < 0.1). In contrast, the difference between male- vs female-headed households for child nutrition outcomes is statistically significant at *P* < 0.05, leading to more confident results in the ‘catch-up’ effects observed amongst female-headed households for these outcomes. As children in female-headed households were found to have a statistically significant nutrition disadvantage at baseline (Table A1c), these findings would suggest that the CGP may have contributed to a reduction in the gendered gap between these two types of households.

## Discussion

CTs have been presented as tools to promote equity amongst children. We tested whether Lesotho’s CGP might have promoted health equity in targeted communities during the early phases of the programme (2011–2013), prior to the introduction of Cash Plus interventions. We have explored whether the CGP had led to a ‘catch-up’ effect and reduced the health gap between beneficiary children and non-beneficiary children (as a whole and within specific subgroups) within 2 years in targeted communities.

### Main findings

Although the programme’s 2014 evaluation had found that the CGP had reduced illnesses amongst beneficiary children, our study shows that this change was not statistically significant, thus rejecting the hypothesis of a catch-up for this outcome. As non-eligible households also reported a slightly higher incidence of child illnesses at baseline, a reduction in inequalities in child morbidity at the community level is unlikely. This suggests that there might be other factors of vulnerability—beyond those identified as selection criteria for the CGP—driving the patterns of childhood illnesses in these communities. Second, our findings do not support the hypothesis that the CGP allowed a reduction in the gap in access to healthcare by making funds available to recipients to cover the cost of transport or medicines. While previous qualitative research had found indications that the CGP improved recipient children’s access to medicines (Kardan, 2014), our findings suggest that these improvements were not enough to overcome the various accessibility and/or affordability barriers to healthcare identified in the baseline study (e.g. distance and transport) (Pellerano *et al.*, 2012). Yet, our robustness test by district suggests that the effect of the CGP on this latter outcome may have been influenced by trends specific to individual districts. As the programme is expanded to further households, an analysis at the district level may help better understand these local trends and the factors that influence them.

Our findings regarding self-assessed health and nutrition outcomes help illustrate a key finding of this study regarding the impact of the CGP on different vulnerable groups. As our subgroup analyses show, the CGP seems to have led to a catch-up effect between eligible and non-eligible in self-assessed child health for beneficiary children who were comparatively more food secure at baseline. A possible explanation for this effect is how the CGP funds were spent. As the 2014 evaluation showed, beneficiary households spent most of their resources on food (Pellerano *et al.*, 2014). Hence, it is possible that more food-secure households had slightly more flexibility to invest CGP funds into a wider range of expenditures, leading to an increased feeling of health and well-being and a larger reduction in stress in these households, as programme stakeholders suggested in our qualitative study (Besnier *et al.*, 2024). Meanwhile, more food-insecure beneficiary households might have had to dedicate a larger portion of this additional income to their children’s basic nutrition needs, especially in the context of poor harvests. However, further analysis of a wider sample would be necessary to confirm this hypothesis.

Our findings on nutrition outcomes further illustrate a differentiated effect of the programme by types of households. While our overall findings did not find a statistically significant reduction in the gap between beneficiary and non-beneficiary children regarding their nutrition, the subgroup analysis by gender of the head of the household suggests that this might be the result of opposite trends between male-headed and female-headed households. Indeed, female-headed households, who tended to be more vulnerable at baseline, did see a catch-up effect in their children’s outcomes further to the CGP. If these findings were to be confirmed as the programme is scaled up to the whole country, they would support the gender equity potential of the CGP for selected outcomes. If so, the results would be consistent with the theory that improvements are linked to selected gendered empowerment processes.

In summary, this study validates some of the effects identified in our conceptual background: the CGP’s catch-up effect in selected subgroups supports a direct effect of the programme on the health gap through the improvement of children’s material circumstances such as access to economic resources (rather than access to healthcare). However, our study does not support a wider direct effect of the CGP in the community via spillover. Finally, the limitations of our datasets prevented us from exploring CGP’s potential indirect effects on the health gap through economic empowerment. Hence, our hypothesis is only partially confirmed.

### Strengths and limitations

Unlike many CT pilots and programmes, the CGP’s monitoring and evaluation included data collection amongst both eligible and non-eligible households, thus offering a unique opportunity to explore the programme’s effect on child health inequalities at community level. The randomized controlled trial design followed by the CGP evaluation in these early phases provides a promising design for exploring the direct effect of the programme on inequalities.

Yet, certain limitations should be noted. First, children under 6 years old make up <12% of the total sample (Pellerano *et al.*, 2012). The small size of our population of interest limited our ability to further explore trends and phenomena at the district levels and may have impacted the statistical power of our analysis for some of the outcomes we studied. Second, this study relies on secondary data analysis. These factors affected our ability to test potential correlations between the CGP’s health equity effect and measures of economic empowerment identified in our qualitative study, as the samples for these indicators would have been too small to lead to meaningful interpretation. Instead, our study focuses on the CGP’s impact on different vulnerable groups. Based on the CGP’s theory of change and the findings from our qualitative study, we assume that if the CGP had led to an empowerment of vulnerable households, comparatively more vulnerable households would have seen a more important catch-up effect (Pellerano *et al.*, 2014; Besnier *et al.*, 2024).

Third, as our study relies on survey data, we cannot exclude that recall bias or a subjective interpretation of health outcomes—such as self-assessed health status or hunger—may have impacted households’ responses. Other objective child health outcomes—such as immunization or anthropometric indicators—could not be included, as they were only collected in the follow-up survey. However, our qualitative study in the E4HE Lesotho project allowed us to contextualize the concept of health in the CGP according to selected stakeholders and helped us select the most relevant variables in the survey data. Future evaluation of the programme may consider crossing survey data with other records (e.g. health facilities records) or further exploring the meaning of health for beneficiaries directly.

Finally, although our study focused on the CGP phases before the introduction of Cash Plus components, we cannot exclude the possibility that other interventions in the targeted areas, such as the 2012–2013 food emergency grant distributed to CGP-eligible households as well as other emergency support available from local authorities and NGOs, may have contributed to the effects we observe here. Hence, while our study does support a catch-up effect of the CGP for female-headed households, the causal link between the CGP and these effects must be interpreted with caution.

This study contributes to the field of CT research in two ways. First, it helps build the evidence base on the effect of CTs like the CGP on the health gap. Previous research had explored the impact of the CGP on different groups of eligible households (heterogeneity analysis) (Pellerano *et al.*, 2014; Sebastian *et al.*, 2017). There is also a growing body of literature exploring the effect of CTs and safety net programmes on various determinants of health for non-eligible households (indirect treatment effect or spillovers on education, consumption or food security) (Angelucci and De Giorgi, 2009; Angelucci *et al.*, 2010; Beegle *et al.*, 2018; Carraro and Ferrone, 2019). For example, previous studies on the CGP had found positive economic spillover and selective food security spillover amongst non-eligible households (Thome *et al.*, 2016; Carraro and Ferrone, 2019). Our study points towards the potential of CT to reduce the health gap amongst specific population subgroups. Second, our subgroup analysis suggests that CTs affect vulnerabilities differently and may not help reduce health disparities across all factors of health inequities. These differences may be rooted in the different needs, preferences, choices or vision of child well-being of various vulnerable groups. For example, Yoong *et al.* (2012) have highlighted how the gender of the adult recipient affected different child outcomes rather than female recipients being systematically more family-friendly. In their study of the psychosocial effects of CTs, Samuels and Stavropoulou (2016) have shown how different factors of vulnerabilities may affect the psychosocial effect of these programmes on various vulnerable groups. This study further supports CTs having differentiated impacts on health inequalities according to the characteristics of the households and the outcomes of choice, possibly because of different constraints, opportunities, preferences or definitions. To better understand how CTs can contribute to child health equity, future research should more systematically explore their impact amongst different population groups and the processes through which these equity effects can be enhanced.

## Conclusion

In this paper, we examined to what extent CT programmes could reduce child health inequalities. First, we explored whether the CGP reduced the gap in selected child health outcomes between beneficiary households and non-beneficiary households in areas receiving the CGP compared with control areas. Second, we examined whether this effect differs according to selected factors of vulnerability, namely child food security at baseline (as a proxy of access to economic resources) and the gender of the head of the household. We find that while the changes observed may suggest a catch-up effect amongst beneficiary households, these effects are not statistically significant. The robustness tests and subgroup analysis show that these overall results may be impacted by opposing local or group-specific trends. Second, we find that the CGP was associated with a reduction in the gap for selected child health outcomes amongst specific subgroups. However, these catch-up effects did not necessarily benefit the comparatively more vulnerable groups across all outcomes. This study highlights the potential of CT programmes like the CGP to address health disparities in preschool children for selected population groups in the community. However, these effects are complex and do not necessarily lead to an overall catch-up effect for beneficiary children.

## Supplementary Material

czad044_Supp

## Data Availability

The data underlying this article were provided by the Transfer Project data portal (hosted by the University of North Carolina at Chapel Hill—Carolina Population Center (UNC CPC) under licence (application number 30032001)). Request for data can be submitted directly to UNC CPC (UNC Carolina Population Center, 2021).

## Footnotes

1. The Proxy Means Testing aimed to assess the household’s level of poverty based on dwelling conditions, household characteristics and ownership of selective assets (Pellerano *et al.*, 2014).

2. Theory of change is an approach designed to support strategic planning and evaluation in development programmes. It can be used as a tool to lay out the logical sequence of a programme to describe how individual activities might lead to the desired change or outcomes and the different pathways through which this change may occur (Vogel, 2012).

3. Due to programme constraints, follow-up data were only collected amongst half of the non-eligible households.

4. The bukana health card is distributed to parents at healthcare facilities in Lesotho and records key child health indicators—such as weight, height and immunization—for birth until 36 months of age (Pellerano *et al.*, 2012b). It can be used as a proxy to assess healthcare utilization.

5. Having a passport allows Basotho adults to seek work in neighbouring countries, a key source of revenues for rural households (Pellerano *et al.*, 2012b; Ministry of Labour and Employment, IOM, 2017).

## Reflexivity statement

The authors’ group includes researchers and practitioners, based in Norway and Lesotho. This article was led by early-career researchers and practitioners, with support from more experienced researchers. The authors also ensure a review and validation of the findings through an internal validation process involving civil servants in charge of the programme of interest, international organizations present in Lesotho and academics with expertise in cash transfers.

## Appendix 1. Baseline balance

**Table A1a.** Baseline balance between eligible and non-eligible households at baseline

	(1)		(2)		*t*-test
	Non-eligible		Eligible		Difference
	*N*	Mean/SE	*N*	Mean/SE	(1)-(2)
Male-headed households	202	0.728	510	0.547	0.181***
		[0.031]		[0.022]	
Adult education achievement	229	0.318	537	0.297	0.021
		[0.015]		[0.009]	
Households own or cultivate land in the past year	229	0.904	537	0.872	0.032
		[0.020]		[0.014]	
Households have access to a piped water supply source	229	0.537	537	0.529	0.008
		[0.033]		[0.022]	
Children <3 years old have a bukana card	229	0.738	537	0.613	0.125***
		[0.029]		[0.021]	
Children in the household have a birth certificate	229	0.188	537	0.143	0.044
		[0.026]		[0.015]	
Has received a CT/public assistance in the last year	229	0.135	536	0.101	0.035
		[0.023]		[0.013]	
Adults in the household have a passport	227	0.700	531	0.467	0.233***
		[0.030]		[0.022]	
Live in treatment areas	229	0.541	537	0.579	−0.038
		[0.033]		[0.021]	
No child illness in the last 30 days	229	0.552	537	0.608	−0.055
		[0.031]		[0.020]	
Healthcare spending on children in the last 3 months	229	0.250	529	0.189	0.061**
		[0.027]		[0.016]	
Self-assessed child health status	225	0.946	533	0.906	0.039*
		[0.014]		[0.012]	
No child going to bed hungry in the last 3 months	229	0.782	537	0.659	0.122***
		[0.027]		[0.020]	
No child eating fewer meals in the last 3 months	229	0.550	537	0.285	0.265***
		[0.033]		[0.019]	
No child eating smaller meals in the last 3 months	229	0.520	537	0.270	0.250***
		[0.033]		[0.019]	

The values displayed for *t*-tests are the differences in the means across the groups.

***, ** and * indicate significance at the 1 and 5% critical levels.

**Table A1b.** Baseline balance between food-insecure and food-secure households at baseline

	(1)		(2)		*t*-test
	Food-insecure households		Food-secure households		Difference
	*N*	Mean/SE	*N*	Mean/SE	(1)-(2)
Male-headed households	460	0.543	252	0.698	−0.155***
		[0.023]		[0.029]	
Adult education achievement	487	0.294	279	0.319	−0.025
		[0.010]		[0.013]	
Households own or cultivate land in the past year	487	0.869	279	0.903	−0.035
		[0.015]		[0.018]	
Households have access to a piped water supply source	487	0.534	279	0.527	0.007
		[0.023]		[0.030]	
Children <3 years old have a bukana card	487	0.643	279	0.663	−0.020
		[0.022]		[0.028]	
Children in the household have a birth certificate	487	0.144	279	0.179	−0.035
		[0.016]		[0.023]	
Has received a CT/public assistance in the last year	486	0.093	279	0.143	−0.051**
		[0.013]		[0.021]	
Adults in the household have a passport	480	0.481	278	0.633	−0.152***
		[0.023]		[0.029]	
Live in treatment areas	487	0.546	279	0.606	−0.060
		[0.023]		[0.029]	
Eligible to CGP	487	0.789	279	0.548	0.240***
		[0.019]		[0.030]	
No child illness in the last 30 days	487	0.557	279	0.651	−0.095***
		[0.021]		[0.027]	
Healthcare spending on children in the last 3 months	483	0.212	275	0.199	0.013
		[0.017]		[0.023]	
Self-assessed child health status	481	0.911	277	0.931	−0.021
		[0.012]		[0.015]	
No child going to bed hungry in the last 3 months	487	0.526	279	0.993	−0.467***
		[0.023]		[0.005]	
No child eating smaller meals in the last 3 months	487	0.023	279	0.907	−0.884***
		[0.007]		[0.017]	

The values displayed for *t*-tests are the differences in the means across the groups.

*** and ** indicate significance at the 1 and 5% critical levels.

**Table A1c.** Baseline balance between female-headed (FHH) and male-headed households (MHH) at baseline

	(1)		(2)		*t*-test
	FHH		MHH		Difference
	*N*	Mean/SE	*N*	Mean/SE	(1)-(2)
Adult education achievement	286	0.339	426	0.271	0.068***
		[0.011]		[0.011]	
Households own or cultivate land in the past year	286	0.857	426	0.897	−0.040
		[0.021]		[0.015]	
Households have access to a piped water supply source	286	0.531	426	0.542	−0.011
		[0.030]		[0.024]	
Children <3 years old have a bukana card	286	0.594	426	0.681	−0.086**
		[0.029]		[0.023]	
Children in the household have a birth certificate	286	0.150	426	0.157	−0.007
		[0.021]		[0.018]	
Has received a CT/public assistance in the last year	285	0.154	426	0.094	0.060**
		[0.021]		[0.014]	
Adults in the household have a passport	282	0.401	424	0.583	−0.182***
		[0.029]		[0.024]	
Live in treatment areas	286	0.531	426	0.594	−0.062*
		[0.030]		[0.024]	
Eligible to CGP	286	0.808	426	0.655	0.153***
		[0.023]		[0.023]	
No child illness in the last 30 days	286	0.593	426	0.595	−0.002
		[0.027]		[0.022]	
Healthcare Spending on children in the last 3 months	284	0.162	420	0.239	−0.077***
		[0.020]		[0.020]	
Self-assessed child health status	282	0.924	422	0.910	0.013
		[0.015]		[0.013]	
No child going to bed hungry in the last 3 months	286	0.692	426	0.702	−0.010
		[0.027]		[0.022]	
No child eating fewer meals in the last 3 months	286	0.266	426	0.413	−0.147***
		[0.026]		[0.024]	
No child eating smaller meals in the last 3 months	286	0.238	426	0.404	−0.166***
		[0.025]		[0.024]	

The values displayed for *t*-tests are the differences in the means across the groups.

***, ** and * indicate significance at the 1, 5 and 10% critical levels.

## Appendix 2. Spillover analysis

**Table A2a.** Difference-in-difference estimates for child-level outcomes among non-eligible households in household-level analysis

	No child illness in the past 30 days	Healthcare spending on children in the past 3 months	Self-assessed child health status	No child going to bed hungry in the past 3 months	No child eating fewer meals in the past 3 months	No child eating smaller meals in the past 3 months
Area_Treat*F-up	0.034	−0.149**	−0.034	0.017	−0.009	0.002
[Spillover]	(0.082)	(0.071)	(0.050)	(0.070)	(0.081)	(0.080)
Observations	458	458	454	457	458	458
*R*-squared	0.007	0.010	0.017	0.003	0.017	0.021

*Note.* Standard errors clustered on households in parentheses.

***p* < 0.05.

**Table A2b.** Baseline balance between non-eligible households in control and treatment areas

	(1) Control		(2) Treatment		*t*-test difference
	***N***	**Mean/SE**	**N**	**Mean/SE**	**(1)-(2)**
Male-headed households	92	0.641	110	0.800	−0.159**
		[0.050]		[0.038]	
Adult education achievement	105	0.351	124	0.290	0.061**
		[0.022]		[0.021]	
Households own or cultivate land in the past year	105	0.905	124	0.903	0.002
		[0.029]		[0.027]	
Households have access to a piped water supply source	105	0.505	124	0.565	−0.060
		[0.049]		[0.045]	
Children <3 years old have a bukana card	105	0.752	124	0.726	0.027
		[0.042]		[0.040]	
Children in the household have a birth certificate	105	0.229	124	0.153	0.075
		[0.041]		[0.032]	
Has received a CT/public assistance in the last year	105	0.105	124	0.161	−0.057
		[0.030]		[0.033]	
Adults in the household have a passport	104	0.692	123	0.707	−0.015
		[0.045]		[0.041]	
No child illness in the last 30 days	105	0.602	124	0.510	0.092
		[0.045]		[0.043]	
Healthcare spending on children in the last 3 months	105	0.196	124	0.295	−0.099*
		[0.037]		[0.040]	
Self-assessed child health status	104	0.949	121	0.944	0.005
		[0.020]		[0.019]	
No child going to bed hungry in the last 3 months	105	0.762	124	0.798	−0.036
		[0.042]		[0.036]	
No child eating fewer meals in the last 3 months	105	0.543	124	0.556	−0.014
		[0.049]		[0.045]	
No child eating smaller meals in the last 3 months	105	0.514	124	0.524	−0.010
		[0.049]		[0.045]	

The values displayed for *t*-tests are the differences in the means across the groups.

** and * indicate significance at the 5 and 10% critical levels.

**Table A2c.** Difference-in-difference estimates for child-level health outcomes amongst non-eligible households in household-level analysis, controlling for the gender of the head of the household and adult education achievement

	(1)	(2)	(3)	(4)	(5)	(6)
	**No child illness in the last 30 days**	**Healthcare spending on children in the last 3 months**	**Self-assessed child health status**	**Not going to bed hungry in the last 3 months**	**Not eating fewer meals in the last 3 months**	**Not eating smaller meals in the last 3 months**
Area_Treat*F-up	0.034	−0.133*	−0.038	0.020	0.008	0.031
	(0.086)	(0.075)	(0.053)	(0.076)	(0.087)	(0.084)
Treatment areas	−0.107	0.088	−0.001	0.031	−0.002	−0.016
	(0.067)	(0.057)	(0.031)	(0.059)	(0.071)	(0.071)
Follow-up	−0.006	0.097*	−0.028	−0.022	−0.130**	−0.163***
	(0.066)	(0.056)	(0.037)	(0.056)	(0.062)	(0.061)
Gender of the head of the households	−0.018 (0.060)	0.064 (0.049)	0.003 (0.030)	0.077 (0.055)	0.142** (0.061)	0.138** (0.059)
Adult education achievement	0.071 (0.111)	−0.054 (0.096)	0.066 (0.065)	0.156* (0.082)	0.212* (0.121)	0.207* (0.122)
Constant	0.600***	0.173***	0.918***	0.661***	0.363***	0.345***
	(0.072)	(0.057)	(0.040)	(0.065)	(0.078)	(0.077)
Observations	404	404	400	403	404	404
*R*-squared	0.012	0.014	0.015	0.017	0.040	0.045

Robust standard errors are in parentheses.

****p* < 0.01, ***p* < 0.05, **p* < 0.1.

The DDD model presented in our article assesses potential spillover in non-eligible households through a difference-in-differences estimate among non-eligible households in treatment and control areas over the intervention period. Table A3.1 reproduces such estimates with an updated number of observations and *R*-squared estimates for non-eligible households alone. We did not replicate the subgroup analyses for this spillover analysis because the sample size for these subgroups among non-eligible households was very small, which would affect the statistical power of the analysis.

As Table A3a shows, there is no statistically significant spillover effect on almost any of the outcomes of interest. This confirms the initial findings from the CGP evaluation, which found no or minor spillovers across the outcomes evaluators tested (Pellerano *et al.*, 2014). The only exception is healthcare spending, where the coefficient suggests a statistically significant negative spillover in treatment areas.

This result is surprising because it goes against the expected spillover (if any) of a CT programme and the programme stakeholders’ perceptions reported in our qualitative study (Besnier *et al.*, 2024).

The sample of non-eligible households was generally balanced (Table A3b). However, controlling for the two household characteristics whose differences were statistically significant at *P *< 0.05 at baseline affects this coefficient and the associated *P*-values (Table A3c). As non-eligible households in the treatment area also had a higher mean healthcare spending for children at baseline, the negative effect captured in this analysis is likely linked to other household characteristics rather than to a spillover of the CGP.

To conclude, this analysis does not support the observations from programme stakeholders that the CGP had a positive spillover effect on non-beneficiary children’s health and nutrition.

## Appendix 3. Robustness test by districts

Outcomes are coded so that a positive score means good nutrition and health in children under 6 years.

**Table A3a.** DDD model results for child-level outcomes in household-level analysis, excluding one district at a time: child illness

No child illness in the last 30 days	Excluding Maseru (1)	Excluding Leribe (2)	Excluding Berea (3)	Excluding Mafeteng (4)	Excluding Qacha’s Nek (5)
Treatment areas	−0.154**	−0.079	−0.049	−0.064	−0.108*
	(0.070)	(0.072)	(0.070)	(0.072)	(0.064)
Eligible	0.048	0.006	0.034	0.018	0.017
	(0.060)	(0.063)	(0.061)	(0.063)	(0.056)
Follow-up	−0.078	−0.032	−0.001	−0.060	−0.045
	(0.071)	(0.077)	(0.070)	(0.073)	(0.064)
Elig*F-up	0.010	0.016	−0.034	−0.023	−0.009
	(0.084)	(0.089)	(0.086)	(0.088)	(0.076)
Area_Treat*F-up	0.089	0.035	0.012	0.009	0.023
	(0.092)	(0.098)	(0.091)	(0.093)	(0.083)
Area_Treat*Eligible	0.125	0.048	0.012	0.035	0.070
	(0.082)	(0.085)	(0.084)	(0.086)	(0.076)
Area_Treat*Eligible*F-up	−0.032	0.042	0.086	0.083	0.057
	(0.110)	(0.115)	(0.111)	(0.113)	(0.100)
Constant	0.598***	0.604***	0.581***	0.615***	0.613***
	(0.050)	(0.052)	(0.050)	(0.052)	(0.046)
Observations	1224	1186	1144	1108	1466
*R*-squared	0.019	0.006	0.005	0.007	0.009

Robust standard errors are in parentheses.

****p* < 0.01, ***p* < 0.05, **p* < 0.1.

**Table A3b.** DDD model results for child-level outcomes in household-level analysis, excluding one district at a time: healthcare spending for children

Healthcare spending on children in the last 3 months	Excluding Maseru (1)	Excluding Leribe (2)	Excluding Berea (3)	Excluding Mafeteng (4)	Excluding Qacha’s Nek (5)
Treatment areas	0.104*	0.131**	0.058	0.094	0.107*
	(0.060)	(0.064)	(0.061)	(0.061)	(0.056)
Eligible	−0.040	0.023	0.000	−0.002	0.008
	(0.048)	(0.052)	(0.052)	(0.051)	(0.046)
Follow-up	0.136**	0.109*	0.065	0.117*	0.115**
	(0.060)	(0.062)	(0.055)	(0.062)	(0.054)
Elig*F-up	−0.079	−0.102	−0.095	−0.071	−0.092
	(0.069)	(0.073)	(0.067)	(0.075)	(0.064)
Area_Treat*F-up	−0.171**	−0.184**	−0.124	−0.123	−0.146**
	(0.078)	(0.082)	(0.078)	(0.085)	(0.073)
Area_Treat*Eligible	−0.102	−0.174**	−0.051	−0.085	−0.121*
	(0.068)	(0.074)	(0.073)	(0.073)	(0.065)
Area_Treat*Eligible*F-up	0.176*	0.193**	0.080	0.063	0.127
	(0.090)	(0.095)	(0.092)	(0.100)	(0.085)
Constant	0.190***	0.196***	0.212***	0.192***	0.191***
	(0.040)	(0.043)	(0.042)	(0.042)	(0.038)
Observations	1218	1179	1140	1101	1458
*R*-squared	0.020	0.013	0.011	0.012	0.012

Robust standard errors are in parentheses.

****p* < 0.01, ***p* < 0.05, **p* < 0.1.

**Table A3c.** DDD model results for child-level outcomes in household-level analysis, excluding one district at a time: self-assessed health status

Self-assessed child health status	Excluding Maseru (1)	Excluding Leribe (2)	Excluding Berea (3)	Excluding Mafeteng (4)	Excluding Qacha’s Nek (5)
Treatment areas	−0.017	−0.003	−0.017	0.013	−0.002
	(0.032)	(0.027)	(0.033)	(0.036)	(0.027)
Eligible	−0.006	−0.040	−0.034	−0.005	−0.019
	(0.027)	(0.028)	(0.031)	(0.034)	(0.026)
Follow-up	−0.065	−0.051	−0.044	−0.028	−0.032
	(0.040)	(0.037)	(0.038)	(0.044)	(0.034)
Elig*F-up	0.022	0.058	0.067	0.010	0.017
	(0.048)	(0.046)	(0.046)	(0.053)	(0.042)
Area_Treat*F-up	−0.020	−0.055	0.005	−0.037	−0.059
	(0.059)	(0.054)	(0.055)	(0.060)	(0.051)
Area_Treat*Eligible	−0.025	−0.007	−0.021	−0.070	−0.040
	(0.041)	(0.038)	(0.045)	(0.047)	(0.036)
Area_Treat*Eligible*F-up	0.052	0.044	−0.006	0.073	0.081
	(0.069)	(0.065)	(0.067)	(0.072)	(0.060)
Constant	0.949***	0.961***	0.948***	0.929***	0.955***
	(0.022)	(0.020)	(0.022)	(0.028)	(0.020)
Observations	1216	1179	1140	1103	1458
*R*-squared	0.010	0.009	0.007	0.007	0.009

Robust standard errors are in parentheses.

****p* < 0.01, ***p* < 0.05, **p* < 0.1.

**Table A3d.** DDD model results for child-level outcomes in household-level analysis, excluding one district at a time: nutrition

Not going to bed hungry in the last 3 months	Excluding Maseru (1)	Excluding Leribe (2)	Excluding Berea (3)	Excluding Mafeteng (4)	Excluding Qacha’s Nek (5)
Treatment areas	0.041	0.042	0.048	0.009	0.042
	(0.061)	(0.066)	(0.061)	(0.064)	(0.057)
Eligible	−0.131**	−0.159**	−0.174***	−0.160***	−0.117**
	(0.058)	(0.062)	(0.060)	(0.061)	(0.054)
Follow-up	−0.035	0.065	−0.024	−0.079	−0.020
	(0.058)	(0.056)	(0.054)	(0.058)	(0.054)
Elig*F-up	0.039	−0.005	0.144**	0.109	0.033
	(0.073)	(0.073)	(0.070)	(0.076)	(0.067)
Area_Treat*F-up	−0.008	−0.034	0.035	0.066	0.027
	(0.079)	(0.082)	(0.076)	(0.083)	(0.073)
Area_Treat*Eligible	0.036	0.046	0.051	0.059	0.013
	(0.076)	(0.082)	(0.078)	(0.081)	(0.071)
Area_Treat*Eligible*F-up	0.061	0.106	−0.018	−0.010	0.060
	(0.098)	(0.101)	(0.096)	(0.104)	(0.090)
Constant	0.767***	0.740***	0.771***	0.776***	0.755***
	(0.046)	(0.050)	(0.046)	(0.048)	(0.044)
Observations	1223	1185	1144	1107	1465
*R*-squared	0.017	0.035	0.034	0.019	0.020

Robust standard errors are in parentheses.

****p* < 0.01, ***p* < 0.05.

**Table A3e.** DDD model results for child-level outcomes in household-level analysis, excluding one district at a time: nutrition (cont.)

Not eating fewer meals in the last 3 months	Excluding Maseru (1)	Excluding Leribe (2)	Excluding Berea (3)	Excluding Mafeteng (4)	Excluding Qacha’s Nek (5)
Treatment areas	0.000	0.067	0.007	−0.037	0.029
	(0.074)	(0.077)	(0.075)	(0.077)	(0.068)
Eligible	−0.300***	−0.274***	−0.383***	−0.303***	−0.287***
	(0.063)	(0.066)	(0.063)	(0.067)	(0.058)
Follow-up	−0.140**	−0.091	−0.157***	−0.105	−0.122**
	(0.062)	(0.069)	(0.055)	(0.071)	(0.060)
Elig*F-up	0.208***	0.184**	0.258***	0.178**	0.192***
	(0.077)	(0.084)	(0.073)	(0.085)	(0.073)
Area_Treat*F-up	−0.017	−0.045	0.024	−0.002	−0.009
	(0.091)	(0.098)	(0.085)	(0.098)	(0.085)
Area_Treat*Eligible	0.096	−0.003	0.097	0.130	0.053
	(0.086)	(0.089)	(0.086)	(0.090)	(0.079)
Area_Treat*Eligible*F-up	0.050	0.096	0.047	0.012	0.063
	(0.109)	(0.117)	(0.106)	(0.117)	(0.102)
Constant	0.535***	0.519***	0.578***	0.553***	0.531***
	(0.054)	(0.057)	(0.054)	(0.057)	(0.051)
Observations	1224	1186	1144	1108	1466
R-squared	0.037	0.043	0.067	0.033	0.040

Robust standard errors are in parentheses.

****p* < 0.01, ***p* < 0.05.

**Table A3f.** DDD model results for child-level outcomes in household-level analysis, excluding one district at a time: nutrition (cont.)

Not eating smaller meals in the last 3 months	Excluding Maseru (1)	Excluding Leribe (2)	Excluding Berea (3)	Excluding Mafeteng (4)	Excluding Qacha’s Nek (5)
Treatment areas	0.015	0.004	0.022	−0.008	0.015
	(0.074)	(0.078)	(0.075)	(0.078)	(0.068)
Eligible	−0.271***	−0.272***	−0.359***	−0.269***	−0.276***
	(0.063)	(0.065)	(0.063)	(0.067)	(0.058)
Follow-up	−0.163***	−0.117*	−0.181***	−0.092	−0.153**
	(0.061)	(0.069)	(0.054)	(0.070)	(0.060)
Elig*F-up	0.194**	0.199**	0.258***	0.150*	0.200***
	(0.075)	(0.083)	(0.071)	(0.085)	(0.073)
Area_Treat*F-up	−0.024	0.014	0.037	−0.037	0.013
	(0.089)	(0.094)	(0.085)	(0.096)	(0.083)
Area_Treat*Eligible	0.062	0.043	0.077	0.108	0.052
	(0.086)	(0.089)	(0.086)	(0.090)	(0.079)
Area_Treat*Eligible*F-up	0.063	0.022	0.013	0.021	0.035
	(0.108)	(0.113)	(0.105)	(0.116)	(0.100)
Constant	0.500***	0.506***	0.542***	0.513***	0.510***
	(0.054)	(0.057)	(0.055)	(0.058)	(0.051)
Observations	1224	1186	1144	1108	1466
*R*-squared	0.034	0.034	0.061	0.028	0.035

Robust standard errors are in parentheses.

****p* < 0.01, ***p* < 0.05, **p* < 0.1.

## Appendix 4. Subgroup analyses

**Table A4a.** DDD model results for child-level outcomes in household-level analysis by food security status at baseline (outcomes are coded so that a positive score means good nutrition and health in children under 6 years)

	Households where children ‘had to’ eat fewer meals in the last 3 months at baseline (0)			Households where children ‘did not’ have to eat fewer meals in the last 3 months at baseline (1)		
	**Illness in the last 30 days**	**Healthcare spending in the last 3 months**	**Self-assessed health status**	**Illness in the last 30 days**	**Healthcare spending in the last 3 months**	**Self-assessed health status**
Area_Treatment/Control	−0.192**	0.165**	−0.062	−0.010	0.052	0.037
	(0.091)	(0.075)	(0.045)	(0.082)	(0.076)	(0.034)
Eligible	0.046	0.063	−0.038	0.041	−0.086	0.003
	(0.076)	(0.055)	(0.031)	(0.086)	(0.070)	(0.044)
Follow-up	0.035	0.198***	−0.096*	−0.106	0.049	−0.000
	(0.090)	(0.066)	(0.052)	(0.086)	(0.077)	(0.044)
Elig*F-up	−0.088	−0.192**	0.092	0.062	0.020	−0.028
	(0.101)	(0.076)	(0.059)	(0.127)	(0.107)	(0.061)
Area_Treat*F-up	0.070	−0.234**	0.115	0.004	−0.093	−0.154**
	(0.115)	(0.096)	(0.074)	(0.112)	(0.101)	(0.065)
Area_Treat*Eligible	0.132	−0.172**	0.030	0.023	−0.050	−0.092
	(0.103)	(0.084)	(0.053)	(0.112)	(0.095)	(0.056)
Area_Treat*Eligible*F-up	0.061	0.205*	−0.110	−0.035	0.076	0.198**
	(0.130)	(0.108)	(0.083)	(0.159)	(0.133)	(0.088)
Constant	0.569***	0.146***	0.961***	0.626***	0.230***	0.941***
	(0.067)	(0.047)	(0.024)	(0.058)	(0.053)	(0.030)
Observations	976	972	970	562	558	560
*R*-squared	0.018	0.014	0.005	0.015	0.016	0.025

Robust standard errors are in parentheses.

****p* < 0.01, ***p* < 0.05, **p*< 0.1.

**Table A4b.** DDD model results for child-level outcomes in household-level analysis by gender of the head of the households (outcomes are coded so that a positive score means good nutrition and health in children under 6 years)

	Female-headed households			Male-headed households		
	**Illness in the last 30 days**	**Healthcare spending in the last 3 months**	**Self-assessed health status**	**Illness in the last 30 days**	**Healthcare spending in the last 3 months**	**Self-assessed health status**
Area_Treatment/Control	−0.056	−0.048	0.057	−0.123	0.126*	−0.015
	(0.126)	(0.087)	(0.035)	(0.079)	(0.071)	(0.040)
Eligible	−0.051	0.021	−0.002	0.085	−0.001	−0.017
	(0.092)	(0.070)	(0.043)	(0.073)	(0.063)	(0.038)
Follow-up	−0.043	0.141*	−0.064	0.014	0.072	−0.008
	(0.103)	(0.083)	(0.059)	(0.085)	(0.073)	(0.046)
Elig*F-up	0.079	−0.084	0.036	−0.158	−0.086	0.009
	(0.118)	(0.098)	(0.070)	(0.104)	(0.087)	(0.057)
Area_Treat*F-up	0.020	−0.005	−0.072	0.027	−0.152	−0.042
	(0.151)	(0.133)	(0.095)	(0.105)	(0.092)	(0.063)
Area_Treat*Eligible	0.089	0.019	−0.104**	0.020	−0.133	−0.026
	(0.141)	(0.099)	(0.049)	(0.096)	(0.085)	(0.053)
Area_Treat*Eligible*F-up	−0.045	−0.101	0.128	0.136	0.210*	0.031
	(0.170)	(0.148)	(0.107)	(0.130)	(0.109)	(0.078)
Constant	0.624***	0.162***	0.943***	0.605***	0.216***	0.941***
	(0.079)	(0.059)	(0.035)	(0.060)	(0.051)	(0.030)
Observations	572	570	568	852	846	848
*R*-squared	0.002	0.026	0.010	0.016	0.011	0.008
	**Going to bed** **hungryin the** **last 3 months**	**Eating fewer** **meals in the last** **3 months**	**Eating smaller** **meals in the** **last 3 months**	**Going to bed** **hungry in the last** **3 months**	**Eating fewer** **meals in the last** **3 months**	**Eating smaller** **meals in the** **last 3 months**
Area_Treatment/Control	0.061	0.000	−0.015	0.033	−0.003	−0.020
	(0.112)	(0.138)	(0.137)	(0.070)	(0.084)	(0.084)
Eligible	−0.134	−0.276***	−0.266***	−0.157**	−0.287***	−0.262***
	(0.090)	(0.095)	(0.094)	(0.072)	(0.077)	(0.078)
Follow-up	−0.152*	−0.091	−0.091	0.051	−0.153**	−0.203***
	(0.088)	(0.109)	(0.109)	(0.070)	(0.075)	(0.071)
Elig*F-up	0.153	0.147	0.167	0.024	0.267***	0.256***
	(0.107)	(0.122)	(0.120)	(0.089)	(0.098)	(0.094)
Area_Treat*F-up	0.061	−0.205	−0.205	−0.030	0.073	0.112
	(0.141)	(0.159)	(0.159)	(0.091)	(0.102)	(0.097)
Area_Treat*Eligible	0.023	0.076	0.080	0.059	0.112	0.115
	(0.128)	(0.148)	(0.146)	(0.092)	(0.101)	(0.102)
Area_Treat*Eligible*F-up	0.031	0.366**	0.304*	0.056	−0.154	−0.168
	(0.162)	(0.177)	(0.175)	(0.116)	(0.130)	(0.127)
Constant	0.758***	0.455***	0.424***	0.763***	0.559***	0.542***
	(0.075)	(0.087)	(0.087)	(0.056)	(0.065)	(0.065)
Observations	572	572	572	851	852	852
*R*-squared	0.027	0.066	0.051	0.027	0.029	0.025

Robust standard errors in parentheses.

****p* < 0.01, ***p* < 0.05, **p* < 0.1.
