# Supplementary material for: Using cash transfers to promote child health equity: an analysis of Lesotho’s Child Grants Program
Source: Health Policy Plan. 2023 Jul 4;39(2):118–37. doi: 10.1093/heapol/czad044 (PMC11020256; doi:10.1093/heapol/czad044)
Supplement: czad044_Supp [file czad044_supp.zip › E4HE Lesotho 3 Quant Annexes Rev2 clean.docx]

Annex 1. Baseline balance

*Table A1a. Baseline balance between eligible and non-eligible households at baseline*

|  | **(1)** | | **(2)** | | **t-test** |
| --- | --- | --- | --- | --- | --- |
|  | **Non-eligible** | | **Eligible** | | **Difference** |
|  | **N** | **Mean/SE** | **N** | **Mean/SE** | **(1)-(2)** |
| **Male-headed households** | 202 | 0.728 | 510 | 0.547 | 0.181*** |
|  |  | [0.031] |  | [0.022] |  |
| **Adult education achievement** | 229 | 0.318 | 537 | 0.297 | 0.021 |
|  |  | [0.015] |  | [0.009] |  |
| **Households own or cultivate land in the past year** | 229 | 0.904 | 537 | 0.872 | 0.032 |
|  |  | [0.020] |  | [0.014] |  |
| **Households have access to a piped water supply source** | 229 | 0.537 | 537 | 0.529 | 0.008 |
|  |  | [0.033] |  | [0.022] |  |
| **Children <3y.o. have a bukana card** | 229 | 0.738 | 537 | 0.613 | 0.125*** |
|  |  | [0.029] |  | [0.021] |  |
| **Children in the Household have a birth certificate** | 229 | 0.188 | 537 | 0.143 | 0.044 |
|  |  | [0.026] |  | [0.015] |  |
| **Has received a cash transfer/public assistance in the last year** | 229 | 0.135 | 536 | 0.101 | 0.035 |
|  |  | [0.023] |  | [0.013] |  |
| **Adults in the Household have a passport** | 227 | 0.700 | 531 | 0.467 | 0.233*** |
|  |  | [0.030] |  | [0.022] |  |
| **Live in treatment areas** | 229 | 0.541 | 537 | 0.579 | -0.038 |
|  |  | [0.033] |  | [0.021] |  |
| **No child illness in the last 30 days** | 229 | 0.552 | 537 | 0.608 | -0.055 |
|  |  | [0.031] |  | [0.020] |  |
| **Healthcare Spending on children in the last 3 months** | 229 | 0.250 | 529 | 0.189 | 0.061** |
|  |  | [0.027] |  | [0.016] |  |
| **Self-assessed child health status** | 225 | 0.946 | 533 | 0.906 | 0.039* |
|  |  | [0.014] |  | [0.012] |  |
| **No child going to bed hungry in the last 3months** | 229 | 0.782 | 537 | 0.659 | 0.122*** |
|  |  | [0.027] |  | [0.020] |  |
| **No child eating fewer meals in the last 3months** | 229 | 0.550 | 537 | 0.285 | 0.265*** |
|  |  | [0.033] |  | [0.019] |  |
| **No child eating smaller meals in the last 3months** | 229 | 0.520 | 537 | 0.270 | 0.250*** |
|  |  | [0.033] |  | [0.019] |  |

The value displayed for t-tests are the differences in the means across the groups.

***, **, and * indicate significance at the 1, 5, and 10 percent critical level.

*Table A1b. Baseline balance between food-insecure and food-secure households at baseline*

|  | **(1)** | | **(2)** | | **t-test** |
| --- | --- | --- | --- | --- | --- |
|  | **Food insecure households** | | **Food Secure households** | | **Difference** |
|  | **N** | **Mean/SE** | **N** | **Mean/SE** | **(1)-(2)** |
| **Male-headed households** | 460 | 0.543 | 252 | 0.698 | -0.155*** |
|  |  | [0.023] |  | [0.029] |  |
| **Adult education achievement** | 487 | 0.294 | 279 | 0.319 | -0.025 |
|  |  | [0.010] |  | [0.013] |  |
| **Households own or cultivate land in the past year** | 487 | 0.869 | 279 | 0.903 | -0.035 |
|  |  | [0.015] |  | [0.018] |  |
| **Households have access to a piped water supply source** | 487 | 0.534 | 279 | 0.527 | 0.007 |
|  |  | [0.023] |  | [0.030] |  |
| **Children <3y.o. have a bukana card** | 487 | 0.643 | 279 | 0.663 | -0.020 |
|  |  | [0.022] |  | [0.028] |  |
| **Children in the Household have a birth certificate** | 487 | 0.144 | 279 | 0.179 | -0.035 |
|  |  | [0.016] |  | [0.023] |  |
| **Has received a cash transfer/public assistance in the last year** | 486 | 0.093 | 279 | 0.143 | -0.051** |
|  |  | [0.013] |  | [0.021] |  |
| **Adults in the Household have a passport** | 480 | 0.481 | 278 | 0.633 | -0.152*** |
|  |  | [0.023] |  | [0.029] |  |
| **Live in treatment areas** | 487 | 0.546 | 279 | 0.606 | -0.060 |
|  |  | [0.023] |  | [0.029] |  |
| **Eligible to CGP** | 487 | 0.789 | 279 | 0.548 | 0.240*** |
|  |  | [0.019] |  | [0.030] |  |
| **No child illness in the last 30 days** | 487 | 0.557 | 279 | 0.651 | -0.095*** |
|  |  | [0.021] |  | [0.027] |  |
| **Healthcare Spending on children in the last 3 months** | 483 | 0.212 | 275 | 0.199 | 0.013 |
|  |  | [0.017] |  | [0.023] |  |
| **Self-assessed child health status** | 481 | 0.911 | 277 | 0.931 | -0.021 |
|  |  | [0.012] |  | [0.015] |  |
| **No child going to bed hungry in the last 3months** | 487 | 0.526 | 279 | 0.993 | -0.467*** |
|  |  | [0.023] |  | [0.005] |  |
| **No child eating smaller meals in the last 3months** | 487 | 0.023 | 279 | 0.907 | -0.884*** |
|  |  | [0.007] |  | [0.017] |  |

The value displayed for t-tests are the differences in the means across the groups.

***, **, and * indicate significance at the 1, 5, and 10 percent critical level.

*Table A1c. Baseline balance between female-headed (FHH) and male-headed households (MHH) at baseline*

|  | **(1)** | | **(2)** | | **t-test** |
| --- | --- | --- | --- | --- | --- |
|  | **FHH** | | **MHH** | | **Difference** |
|  | **N** | **Mean/SE** | **N** | **Mean/SE** | **(1)-(2)** |
| **Adult education achievement** | 286 | 0.339 | 426 | 0.271 | 0.068*** |
|  |  | [0.011] |  | [0.011] |  |
| **Households own or cultivate land in the past year** | 286 | 0.857 | 426 | 0.897 | -0.040 |
|  |  | [0.021] |  | [0.015] |  |
| **Households have access to a piped water supply source** | 286 | 0.531 | 426 | 0.542 | -0.011 |
|  |  | [0.030] |  | [0.024] |  |
| **Children <3y.o. have a bukana card** | 286 | 0.594 | 426 | 0.681 | -0.086** |
|  |  | [0.029] |  | [0.023] |  |
| **Children in the Household have a birth certificate** | 286 | 0.150 | 426 | 0.157 | -0.007 |
|  |  | [0.021] |  | [0.018] |  |
| **Has received a cash transfer/public assistance in the last year** | 285 | 0.154 | 426 | 0.094 | 0.060** |
|  |  | [0.021] |  | [0.014] |  |
| **Adults in the Household have a passport** | 282 | 0.401 | 424 | 0.583 | -0.182*** |
|  |  | [0.029] |  | [0.024] |  |
| **Live in treatment areas** | 286 | 0.531 | 426 | 0.594 | -0.062* |
|  |  | [0.030] |  | [0.024] |  |
| **Eligible to CGP** | 286 | 0.808 | 426 | 0.655 | 0.153*** |
|  |  | [0.023] |  | [0.023] |  |
| **No child illness in the last 30 days** | 286 | 0.593 | 426 | 0.595 | -0.002 |
|  |  | [0.027] |  | [0.022] |  |
| **Healthcare Spending on children in the last 3 months** | 284 | 0.162 | 420 | 0.239 | -0.077*** |
|  |  | [0.020] |  | [0.020] |  |
| **Self-assessed child health status** | 282 | 0.924 | 422 | 0.910 | 0.013 |
|  |  | [0.015] |  | [0.013] |  |
| **No child going to bed hungry in the last 3months** | 286 | 0.692 | 426 | 0.702 | -0.010 |
|  |  | [0.027] |  | [0.022] |  |
| **No child eating fewer meals in the last 3months** | 286 | 0.266 | 426 | 0.413 | -0.147*** |
|  |  | [0.026] |  | [0.024] |  |
| **No child eating smaller meals in the last 3months** | 286 | 0.238 | 426 | 0.404 | -0.166*** |
|  |  | [0.025] |  | [0.024] |  |

The value displayed for t-tests are the differences in the means across the groups.

***, **, and * indicate significance at the 1, 5, and 10 percent critical level.

# Annex 2. Spillover analysis

The DDD model presented in our article assesses potential spillover in non-eligible households, through a difference-in-differences estimate among noneligible households in treatment and control areas over the intervention period. Table A3.1 reproduces such estimates with updated number of observations and R-squared estimate for non-eligible households alone. We didn’t replicate the subgroup analyses for this spillover analysis because the sample size for these subgroups among noneligible households was very small, which would affect the statistical power of the analysis.

As Table A3a shows, there is no statistically significant spillover effect on almost any of the outcomes of interest. This confirms the initial findings from the CGP evaluation, which found no or minor spillovers across the outcomes evaluators tested (Pellerano *et al.*, 2014). The only exception is healthcare spending, where the coefficient suggests a statistically significant negative spillover in treatment areas.

This result is surprising because it goes against the expected spillover (if any) of a CT program and the program stakeholders’ perceptions reported in our qualitative study (*[Author anonymized]*, Under review).

**Table** A2a. Difference-in-Difference Estimates for Child-Level Outcomes Among Non-Eligible Households in Household-Level Analysis

|  | **No Child Illness in Past 30 Days** | **Healthcare Spending on Children in Past Three Months** | **Self-Assessed Child Health Status** | **No Child Going to Bed Hungry in Past Three Months** | **No Child Eating Fewer Meals in Past Three Months** | **No Child Eating Smaller Meals in Past Three Months** |
| --- | --- | --- | --- | --- | --- | --- |
| **Area_Treat*F-up** | 0.034 | -0.149** | -0.034 | 0.017 | -0.009 | 0.002 |
| **[Spillover]** | (0.082) | (0.071) | (0.050) | (0.070) | (0.081) | (0.080) |
| **Observations** | 458 | 458 | 454 | 457 | 458 | 458 |
| **R-squared** | 0.007 | 0.010 | 0.017 | 0.003 | 0.017 | 0.021 |
| *Note.* Standard errors clustered on household in parentheses  *** p<0.01, ** p<0.05, * p<0.1 | | | | | | |

The sample of noneligible households was generally balanced (see Table A3b). However, controlling for the two household characteristics whose differences were statistically significant at *p* < 0.05 at baseline affects this coefficient and the associated *p* value (see Table A3c). As noneligible households in the treatment area also had a higher mean healthcare spending for children at baseline, the negative effect captured in this analysis is likely linked to other household characteristics rather than to a spillover of the CGP.

Table A2b. Baseline Balance Between Non-Eligible Households in Control and Treatment Areas

|  | **(1)**  **Control** | | **(2)**  **Treatment** | | **t-test Difference** |
| --- | --- | --- | --- | --- | --- |
|  | **N** | **Mean/SE** | **N** | **Mean/SE** | **(1)-(2)** |
| **Male-headed households** | 92 | 0.641 | 110 | 0.800 | -0.159** |
|  |  | [0.050] |  | [0.038] |  |
| **Adult education achievement** | 105 | 0.351 | 124 | 0.290 | 0.061** |
|  |  | [0.022] |  | [0.021] |  |
| **Households own or cultivate land in the past year** | 105 | 0.905 | 124 | 0.903 | 0.002 |
|  |  | [0.029] |  | [0.027] |  |
| **Households have access to a piped water supply source** | 105 | 0.505 | 124 | 0.565 | -0.060 |
|  |  | [0.049] |  | [0.045] |  |
| **Children <3 years old have a bukana card** | 105 | 0.752 | 124 | 0.726 | 0.027 |
|  |  | [0.042] |  | [0.040] |  |
| **Children in the household have a birth certificate** | 105 | 0.229 | 124 | 0.153 | 0.075 |
|  |  | [0.041] |  | [0.032] |  |
| **Has received a cash transfer/public assistance in the last year** | 105 | 0.105 | 124 | 0.161 | -0.057 |
|  |  | [0.030] |  | [0.033] |  |
| **Adults in the household have a passport** | 104 | 0.692 | 123 | 0.707 | -0.015 |
|  |  | [0.045] |  | [0.041] |  |
| **No child illness in the last 30 days** | 105 | 0.602 | 124 | 0.510 | 0.092 |
|  |  | [0.045] |  | [0.043] |  |
| **Healthcare spending on children in the last 3 months** | 105 | 0.196 | 124 | 0.295 | -0.099* |
|  |  | [0.037] |  | [0.040] |  |
| **Self-assessed child health status** | 104 | 0.949 | 121 | 0.944 | 0.005 |
|  |  | [0.020] |  | [0.019] |  |
| **No child going to bed hungry in the last 3months** | 105 | 0.762 | 124 | 0.798 | -0.036 |
|  |  | [0.042] |  | [0.036] |  |
| **No child eating fewer meals in the last 3 months** | 105 | 0.543 | 124 | 0.556 | -0.014 |
|  |  | [0.049] |  | [0.045] |  |
| **No child eating smaller meals in the last 3 months** | 105 | 0.514 | 124 | 0.524 | -0.010 |
|  |  | [0.049] |  | [0.045] |  |
| The value displayed for t-tests are the differences in the means across the groups.  ***, **, and * indicate significance at the 1, 5, and 10 percent critical level. | | | | | |

Table A2c Difference-in-Difference Estimates for Child-Level Health Outcomes Amongst Noneligible Households in Household-Level Analysis, Controlling for the Gender of the Head of the Household and Adult Education Achievement.

|  | (1) | (2) | (3) | (4) | (5) | (6) |
| --- | --- | --- | --- | --- | --- | --- |
|  | No child illness in the last 30 days | Healthcare Spending on children in the last 3 months | Self-assessed child health status | Not going to bed hungry in the last 3months | Not eating fewer meals in the last 3months | Not eating smaller meals in the last 3months |
| Area_Treat*F-up | 0.034 | -0.133* | -0.038 | 0.020 | 0.008 | 0.031 |
|  | (0.086) | (0.075) | (0.053) | (0.076) | (0.087) | (0.084) |
| Treatment areas | -0.107 | 0.088 | -0.001 | 0.031 | -0.002 | -0.016 |
|  | (0.067) | (0.057) | (0.031) | (0.059) | (0.071) | (0.071) |
| Follow-up | -0.006 | 0.097* | -0.028 | -0.022 | -0.130** | -0.163*** |
|  | (0.066) | (0.056) | (0.037) | (0.056) | (0.062) | (0.061) |
| Gender of the head of the Households | -0.018 | 0.064 | 0.003 | 0.077 | 0.142** | 0.138** |
|  | (0.060) | (0.049) | (0.030) | (0.055) | (0.061) | (0.059) |
| Adult education achievement | 0.071 | -0.054 | 0.066 | 0.156* | 0.212* | 0.207* |
|  | (0.111) | (0.096) | (0.065) | (0.082) | (0.121) | (0.122) |
| Constant | 0.600*** | 0.173*** | 0.918*** | 0.661*** | 0.363*** | 0.345*** |
|  | (0.072) | (0.057) | (0.040) | (0.065) | (0.078) | (0.077) |
| Observations | 404 | 404 | 400 | 403 | 404 | 404 |
| R-squared | 0.012 | 0.014 | 0.015 | 0.017 | 0.040 | 0.045 |

Robust standard errors in parentheses

*** p<0.01, ** p<0.05, * p<0.1

To conclude, this analysis does not support the observations from program stakeholders that the CGP had a positive spillover effect on nonbeneficiary children’s health and nutrition.

Annex 3. Robustness test by districts

Outcomes are coded so that positive score means good nutrition and health in children under 6 years

*Table A3a DDD model results for child-level outcomes in household-level analysis, excluding one district at a time: Child illness*

| **No child illness in the last 30 days** | **Excluding Maseru**  **(1)** | **Excluding Leribe**  **(2)** | **Excluding Berea**  **(3)** | **Excluding Mafeteng**  **(4)** | **Excluding Qacha’s Nek**  **(5)** |
| --- | --- | --- | --- | --- | --- |
|  |  |  |  |  |  |
| **Treatment areas** | -0.154** | -0.079 | -0.049 | -0.064 | -0.108* |
|  | (0.070) | (0.072) | (0.070) | (0.072) | (0.064) |
| **Eligible** | 0.048 | 0.006 | 0.034 | 0.018 | 0.017 |
|  | (0.060) | (0.063) | (0.061) | (0.063) | (0.056) |
| **Follow-up** | -0.078 | -0.032 | -0.001 | -0.060 | -0.045 |
|  | (0.071) | (0.077) | (0.070) | (0.073) | (0.064) |
| **Elig*F-up** | 0.010 | 0.016 | -0.034 | -0.023 | -0.009 |
|  | (0.084) | (0.089) | (0.086) | (0.088) | (0.076) |
| **Area_Treat*F-up** | 0.089 | 0.035 | 0.012 | 0.009 | 0.023 |
|  | (0.092) | (0.098) | (0.091) | (0.093) | (0.083) |
| **Area_Treat*Eligible** | 0.125 | 0.048 | 0.012 | 0.035 | 0.070 |
|  | (0.082) | (0.085) | (0.084) | (0.086) | (0.076) |
| **Area_Treat*Eligible*F-up** | -0.032 | 0.042 | 0.086 | 0.083 | 0.057 |
|  | (0.110) | (0.115) | (0.111) | (0.113) | (0.100) |
| **Constant** | 0.598*** | 0.604*** | 0.581*** | 0.615*** | 0.613*** |
|  | (0.050) | (0.052) | (0.050) | (0.052) | (0.046) |
|  |  |  |  |  |  |
| **Observations** | 1,224 | 1,186 | 1,144 | 1,108 | 1,466 |
| **R-squared** | 0.019 | 0.006 | 0.005 | 0.007 | 0.009 |

Robust standard errors in parentheses

*** p<0.01, ** p<0.05, * p<0.1

*Table A3b. DDD model results for child-level outcomes in household-level analysis, excluding one district at a time: Healthcare spending for children*

| **Healthcare Spending on children in the last 3 months** | **Excluding Maseru**  **(1)** | **Excluding Leribe**  **(2)** | **Excluding Berea**  **(3)** | **Excluding Mafeteng**  **(4)** | **Excluding Qacha’s Nek**  **(5)** |
| --- | --- | --- | --- | --- | --- |
| **Treatment areas** | 0.104* | 0.131** | 0.058 | 0.094 | 0.107* |
|  | (0.060) | (0.064) | (0.061) | (0.061) | (0.056) |
| **Eligible** | -0.040 | 0.023 | 0.000 | -0.002 | 0.008 |
|  | (0.048) | (0.052) | (0.052) | (0.051) | (0.046) |
| **Follow-up** | 0.136** | 0.109* | 0.065 | 0.117* | 0.115** |
|  | (0.060) | (0.062) | (0.055) | (0.062) | (0.054) |
| **Elig*F-up** | -0.079 | -0.102 | -0.095 | -0.071 | -0.092 |
|  | (0.069) | (0.073) | (0.067) | (0.075) | (0.064) |
| **Area_Treat*F-up** | -0.171** | -0.184** | -0.124 | -0.123 | -0.146** |
|  | (0.078) | (0.082) | (0.078) | (0.085) | (0.073) |
| **Area_Treat*Eligible** | -0.102 | -0.174** | -0.051 | -0.085 | -0.121* |
|  | (0.068) | (0.074) | (0.073) | (0.073) | (0.065) |
| **Area_Treat*Eligible*F-up** | 0.176* | 0.193** | 0.080 | 0.063 | 0.127 |
|  | (0.090) | (0.095) | (0.092) | (0.100) | (0.085) |
| **Constant** | 0.190*** | 0.196*** | 0.212*** | 0.192*** | 0.191*** |
|  | (0.040) | (0.043) | (0.042) | (0.042) | (0.038) |
|  |  |  |  |  |  |
| **Observations** | 1,218 | 1,179 | 1,140 | 1,101 | 1,458 |
| **R-squared** | 0.020 | 0.013 | 0.011 | 0.012 | 0.012 |

Robust standard errors in parentheses

*** p<0.01, ** p<0.05, * p<0.1

*Table A3c. DDD model results for child-level outcomes in household-level analysis, excluding one district at a time: Self-assessed health status*

| **Self-assessed child health status** | **Excluding Maseru**  **(1)** | **Excluding Leribe**  **(2)** | **Excluding Berea**  **(3)** | **Excluding Mafeteng**  **(4)** | **Excluding Qacha’s Nek**  **(5)** |
| --- | --- | --- | --- | --- | --- |
| **Treatment areas** | -0.017 | -0.003 | -0.017 | 0.013 | -0.002 |
|  | (0.032) | (0.027) | (0.033) | (0.036) | (0.027) |
| **Eligible** | -0.006 | -0.040 | -0.034 | -0.005 | -0.019 |
|  | (0.027) | (0.028) | (0.031) | (0.034) | (0.026) |
| **Follow-up** | -0.065 | -0.051 | -0.044 | -0.028 | -0.032 |
|  | (0.040) | (0.037) | (0.038) | (0.044) | (0.034) |
| **Elig*F-up** | 0.022 | 0.058 | 0.067 | 0.010 | 0.017 |
|  | (0.048) | (0.046) | (0.046) | (0.053) | (0.042) |
| **Area_Treat*F-up** | -0.020 | -0.055 | 0.005 | -0.037 | -0.059 |
|  | (0.059) | (0.054) | (0.055) | (0.060) | (0.051) |
| **Area_Treat*Eligible** | -0.025 | -0.007 | -0.021 | -0.070 | -0.040 |
|  | (0.041) | (0.038) | (0.045) | (0.047) | (0.036) |
| **Area_Treat*Eligible*F-up** | 0.052 | 0.044 | -0.006 | 0.073 | 0.081 |
|  | (0.069) | (0.065) | (0.067) | (0.072) | (0.060) |
| **Constant** | 0.949*** | 0.961*** | 0.948*** | 0.929*** | 0.955*** |
|  | (0.022) | (0.020) | (0.022) | (0.028) | (0.020) |
|  |  |  |  |  |  |
| **Observations** | 1,216 | 1,179 | 1,140 | 1,103 | 1,458 |
| **R-squared** | 0.010 | 0.009 | 0.007 | 0.007 | 0.009 |

Robust standard errors in parentheses

*** p<0.01, ** p<0.05, * p<0.1

*Table A3d. DDD model results for child-level outcomes in household-level analysis, excluding one district at a time: Nutrition*

| **Not going to bed hungry in the last 3months** | **Excluding Maseru**  **(1)** | **Excluding Leribe**  **(2)** | **Excluding Berea**  **(3)** | **Excluding Mafeteng**  **(4)** | **Excluding Qacha’s Nek**  **(5)** |
| --- | --- | --- | --- | --- | --- |
| **Treatment areas** | 0.041 | 0.042 | 0.048 | 0.009 | 0.042 |
|  | (0.061) | (0.066) | (0.061) | (0.064) | (0.057) |
| **Eligible** | -0.131** | -0.159** | -0.174*** | -0.160*** | -0.117** |
|  | (0.058) | (0.062) | (0.060) | (0.061) | (0.054) |
| **Follow-up** | -0.035 | 0.065 | -0.024 | -0.079 | -0.020 |
|  | (0.058) | (0.056) | (0.054) | (0.058) | (0.054) |
| **Elig*F-up** | 0.039 | -0.005 | 0.144** | 0.109 | 0.033 |
|  | (0.073) | (0.073) | (0.070) | (0.076) | (0.067) |
| **Area_Treat*F-up** | -0.008 | -0.034 | 0.035 | 0.066 | 0.027 |
|  | (0.079) | (0.082) | (0.076) | (0.083) | (0.073) |
| **Area_Treat*Eligible** | 0.036 | 0.046 | 0.051 | 0.059 | 0.013 |
|  | (0.076) | (0.082) | (0.078) | (0.081) | (0.071) |
| **Area_Treat*Eligible*F-up** | 0.061 | 0.106 | -0.018 | -0.010 | 0.060 |
|  | (0.098) | (0.101) | (0.096) | (0.104) | (0.090) |
| **Constant** | 0.767*** | 0.740*** | 0.771*** | 0.776*** | 0.755*** |
|  | (0.046) | (0.050) | (0.046) | (0.048) | (0.044) |
|  |  |  |  |  |  |
| **Observations** | 1,223 | 1,185 | 1,144 | 1,107 | 1,465 |
| **R-squared** | 0.017 | 0.035 | 0.034 | 0.019 | 0.020 |

Robust standard errors in parentheses

*** p<0.01, ** p<0.05, * p<0.1

*Table A3e. DDD model results for child-level outcomes in household-level analysis, excluding one district at a time: Nutrition (cont.)*

| **Not eating fewer meals in the last 3months** | **Excluding Maseru**  **(1)** | **Excluding Leribe**  **(2)** | **Excluding Berea**  **(3)** | **Excluding Mafeteng**  **(4)** | **Excluding Qacha’s Nek**  **(5)** |
| --- | --- | --- | --- | --- | --- |
| **Treatment areas** | 0.000 | 0.067 | 0.007 | -0.037 | 0.029 |
|  | (0.074) | (0.077) | (0.075) | (0.077) | (0.068) |
| **Eligible** | -0.300*** | -0.274*** | -0.383*** | -0.303*** | -0.287*** |
|  | (0.063) | (0.066) | (0.063) | (0.067) | (0.058) |
| **Follow-up** | -0.140** | -0.091 | -0.157*** | -0.105 | -0.122** |
|  | (0.062) | (0.069) | (0.055) | (0.071) | (0.060) |
| **Elig*F-up** | 0.208*** | 0.184** | 0.258*** | 0.178** | 0.192*** |
|  | (0.077) | (0.084) | (0.073) | (0.085) | (0.073) |
| **Area_Treat*F-up** | -0.017 | -0.045 | 0.024 | -0.002 | -0.009 |
|  | (0.091) | (0.098) | (0.085) | (0.098) | (0.085) |
| **Area_Treat*Eligible** | 0.096 | -0.003 | 0.097 | 0.130 | 0.053 |
|  | (0.086) | (0.089) | (0.086) | (0.090) | (0.079) |
| **Area_Treat*Eligible*F-up** | 0.050 | 0.096 | 0.047 | 0.012 | 0.063 |
|  | (0.109) | (0.117) | (0.106) | (0.117) | (0.102) |
| **Constant** | 0.535*** | 0.519*** | 0.578*** | 0.553*** | 0.531*** |
|  | (0.054) | (0.057) | (0.054) | (0.057) | (0.051) |
|  |  |  |  |  |  |
| **Observations** | 1,224 | 1,186 | 1,144 | 1,108 | 1,466 |
| **R-squared** | 0.037 | 0.043 | 0.067 | 0.033 | 0.040 |

Robust standard errors in parentheses

*** p<0.01, ** p<0.05, * p<0.1

*Table A3f. DDD model results for child-level outcomes in household-level analysis, excluding one district at a time: Nutrition (cont.)*

| **Not eating smaller meals in the last 3months** | **Excluding Maseru**  **(1)** | **Excluding Leribe**  **(2)** | **Excluding Berea**  **(3)** | **Excluding Mafeteng**  **(4)** | **Excluding Qacha’s Nek**  **(5)** |
| --- | --- | --- | --- | --- | --- |
| **Treatment areas** | 0.015 | 0.004 | 0.022 | -0.008 | 0.015 |
|  | (0.074) | (0.078) | (0.075) | (0.078) | (0.068) |
| **Eligible** | -0.271*** | -0.272*** | -0.359*** | -0.269*** | -0.276*** |
|  | (0.063) | (0.065) | (0.063) | (0.067) | (0.058) |
| **Follow-up** | -0.163*** | -0.117* | -0.181*** | -0.092 | -0.153** |
|  | (0.061) | (0.069) | (0.054) | (0.070) | (0.060) |
| **Elig*F-up** | 0.194** | 0.199** | 0.258*** | 0.150* | 0.200*** |
|  | (0.075) | (0.083) | (0.071) | (0.085) | (0.073) |
| **Area_Treat*F-up** | -0.024 | 0.014 | 0.037 | -0.037 | 0.013 |
|  | (0.089) | (0.094) | (0.085) | (0.096) | (0.083) |
| **Area_Treat*Eligible** | 0.062 | 0.043 | 0.077 | 0.108 | 0.052 |
|  | (0.086) | (0.089) | (0.086) | (0.090) | (0.079) |
| **Area_Treat*Eligible*F-up** | 0.063 | 0.022 | 0.013 | 0.021 | 0.035 |
|  | (0.108) | (0.113) | (0.105) | (0.116) | (0.100) |
| **Constant** | 0.500*** | 0.506*** | 0.542*** | 0.513*** | 0.510*** |
|  | (0.054) | (0.057) | (0.055) | (0.058) | (0.051) |
|  |  |  |  |  |  |
| **Observations** | 1,224 | 1,186 | 1,144 | 1,108 | 1,466 |
| **R-squared** | 0.034 | 0.034 | 0.061 | 0.028 | 0.035 |

Robust standard errors in parentheses

*** p<0.01, ** p<0.05, * p<0.1

Annex 4. Subgroup analyses

*Table A4a. DDD model results for child-level outcomes in household-level analysis by food security status at baseline.*

Outcomes are coded so that positive score means good nutrition and health in children under 6 years

|  | Households where children **had to** eat fewer meals in the last 3 months at baseline (0) | | | Households where children **did not** have to eat fewer meals in the last 3 months at baseline (1) | | |
| --- | --- | --- | --- | --- | --- | --- |
|  | **Illness in the last 30 days** | **Healthcare Spending in the last 3 months** | **Self-assessed health status** | **Illness in the last 30 days** | **Healthcare Spending in the last 3 months** | **Self-assessed health status** |
| **Area_Treatment/Control** | -0.192** | 0.165** | -0.062 | -0.010 | 0.052 | 0.037 |
|  | (0.091) | (0.075) | (0.045) | (0.082) | (0.076) | (0.034) |
| **Eligible** | 0.046 | 0.063 | -0.038 | 0.041 | -0.086 | 0.003 |
|  | (0.076) | (0.055) | (0.031) | (0.086) | (0.070) | (0.044) |
| **Follow-up** | 0.035 | 0.198*** | -0.096* | -0.106 | 0.049 | -0.000 |
|  | (0.090) | (0.066) | (0.052) | (0.086) | (0.077) | (0.044) |
| **Elig*F-up** | -0.088 | -0.192** | 0.092 | 0.062 | 0.020 | -0.028 |
|  | (0.101) | (0.076) | (0.059) | (0.127) | (0.107) | (0.061) |
| **Area_Treat*F-up** | 0.070 | -0.234** | 0.115 | 0.004 | -0.093 | -0.154** |
|  | (0.115) | (0.096) | (0.074) | (0.112) | (0.101) | (0.065) |
| **Area_Treat*Eligible** | 0.132 | -0.172** | 0.030 | 0.023 | -0.050 | -0.092 |
|  | (0.103) | (0.084) | (0.053) | (0.112) | (0.095) | (0.056) |
| **Area_Treat*Eligible*F-up** | 0.061 | 0.205* | -0.110 | -0.035 | 0.076 | 0.198** |
|  | (0.130) | (0.108) | (0.083) | (0.159) | (0.133) | (0.088) |
| **Constant** | 0.569*** | 0.146*** | 0.961*** | 0.626*** | 0.230*** | 0.941*** |
|  | (0.067) | (0.047) | (0.024) | (0.058) | (0.053) | (0.030) |
|  |  |  |  |  |  |  |
| **Observations** | 976 | 972 | 970 | 562 | 558 | 560 |
| **R-squared** | 0.018 | 0.014 | 0.005 | 0.015 | 0.016 | 0.025 |

Robust standard errors in parentheses

*** p<0.01, ** p<0.05, * p<0.1

*Table A4b. DDD model results for child-level outcomes in household-level analysis by gender of the head of the households.*

Outcomes are coded so that positive score means good nutrition and health in children under 6 years

|  | **Female-headed households** | | | **Male-headed households** | | |
| --- | --- | --- | --- | --- | --- | --- |
|  | **Illness in the last 30 days** | **Healthcare Spending in the last 3 months** | **Self-assessed health status** | **Illness in the last 30 days** | **Healthcare Spending in the last 3 months** | **Self-assessed health status** |
| **Area_Treatment/Control** | -0.056 | -0.048 | 0.057 | -0.123 | 0.126* | -0.015 |
|  | (0.126) | (0.087) | (0.035) | (0.079) | (0.071) | (0.040) |
| **Eligible** | -0.051 | 0.021 | -0.002 | 0.085 | -0.001 | -0.017 |
|  | (0.092) | (0.070) | (0.043) | (0.073) | (0.063) | (0.038) |
| **Follow-up** | -0.043 | 0.141* | -0.064 | 0.014 | 0.072 | -0.008 |
|  | (0.103) | (0.083) | (0.059) | (0.085) | (0.073) | (0.046) |
| **Elig*F-up** | 0.079 | -0.084 | 0.036 | -0.158 | -0.086 | 0.009 |
|  | (0.118) | (0.098) | (0.070) | (0.104) | (0.087) | (0.057) |
| **Area_Treat*F-up** | 0.020 | -0.005 | -0.072 | 0.027 | -0.152 | -0.042 |
|  | (0.151) | (0.133) | (0.095) | (0.105) | (0.092) | (0.063) |
| **Area_Treat*Eligible** | 0.089 | 0.019 | -0.104** | 0.020 | -0.133 | -0.026 |
|  | (0.141) | (0.099) | (0.049) | (0.096) | (0.085) | (0.053) |
| **Area_Treat*Eligible*F-up** | -0.045 | -0.101 | 0.128 | 0.136 | 0.210* | 0.031 |
|  | (0.170) | (0.148) | (0.107) | (0.130) | (0.109) | (0.078) |
| **Constant** | 0.624*** | 0.162*** | 0.943*** | 0.605*** | 0.216*** | 0.941*** |
|  | (0.079) | (0.059) | (0.035) | (0.060) | (0.051) | (0.030) |
|  |  |  |  |  |  |  |
| **Observations** | 572 | 570 | 568 | 852 | 846 | 848 |
| **R-squared** | 0.002 | 0.026 | 0.010 | 0.016 | 0.011 | 0.008 |

*Table A4b. (cont.)*

|  | **Female-headed households** | | | **Male-headed households** | | |
| --- | --- | --- | --- | --- | --- | --- |
|  | **Going to bed hungry in the last 3months** | **Eating fewer meals in the last 3months** | **Eating smaller meals in the last 3months** | **Going to bed hungry in the last 3months** | **Eating fewer meals in the last 3months** | **Eating smaller meals in the last 3months** |
| **Area_Treatment/Control** | 0.061 | 0.000 | -0.015 | 0.033 | -0.003 | -0.020 |
|  | (0.112) | (0.138) | (0.137) | (0.070) | (0.084) | (0.084) |
| **Eligible** | -0.134 | -0.276*** | -0.266*** | -0.157** | -0.287*** | -0.262*** |
|  | (0.090) | (0.095) | (0.094) | (0.072) | (0.077) | (0.078) |
| **Follow-up** | -0.152* | -0.091 | -0.091 | 0.051 | -0.153** | -0.203*** |
|  | (0.088) | (0.109) | (0.109) | (0.070) | (0.075) | (0.071) |
| **Elig*F-up** | 0.153 | 0.147 | 0.167 | 0.024 | 0.267*** | 0.256*** |
|  | (0.107) | (0.122) | (0.120) | (0.089) | (0.098) | (0.094) |
| **Area_Treat*F-up** | 0.061 | -0.205 | -0.205 | -0.030 | 0.073 | 0.112 |
|  | (0.141) | (0.159) | (0.159) | (0.091) | (0.102) | (0.097) |
| **Area_Treat*Eligible** | 0.023 | 0.076 | 0.080 | 0.059 | 0.112 | 0.115 |
|  | (0.128) | (0.148) | (0.146) | (0.092) | (0.101) | (0.102) |
| **Area_Treat*Eligible*F-up** | 0.031 | 0.366** | 0.304* | 0.056 | -0.154 | -0.168 |
|  | (0.162) | (0.177) | (0.175) | (0.116) | (0.130) | (0.127) |
| **Constant** | 0.758*** | 0.455*** | 0.424*** | 0.763*** | 0.559*** | 0.542*** |
|  | (0.075) | (0.087) | (0.087) | (0.056) | (0.065) | (0.065) |
|  |  |  |  |  |  |  |
| **Observations** | 572 | 572 | 572 | 851 | 852 | 852 |
| **R-squared** | 0.027 | 0.066 | 0.051 | 0.027 | 0.029 | 0.025 |
